# An Efficient Strategy for Electroreduction Reactor Outlet Fractioning into Valuable Products

**DOI:** 10.1021/acs.iecr.3c00090

**Published:** 2023-05-26

**Authors:** Mariana C.N. Bessa, Azahara Luna-Triguero, Jose M. Vicent-Luna, Paulo M.O.C. Carmo, Mihalis N. Tsampas, Ana Mafalda Ribeiro, Alírio E. Rodrigues, Sofia Calero, Alexandre F.P. Ferreira

**Affiliations:** † Laboratory of Separation and Reaction Engineering−Laboratory of Catalysis and Materials (LSRE-LCM), Department of Chemical Engineering, University of Porto, Rua Dr. Roberto Frias, s/n, 4200-465 Porto, Portugal; ‡ ALiCE−Associate Laboratory in Chemical Engineering, Faculty of Engineering, University of Porto, Rua Dr. Roberto Frias, 4200-465 Porto, Portugal; § Energy Technology, Department of Mechanical Engineering, Eindhoven University of Technology, P.O. Box 513, 5600 MB Eindhoven, The Netherlands; ∥ Eindhoven Institute for Renewable Energy Systems (EIRES), Eindhoven University of Technology, P.O. Box 513, 5600 MB Eindhoven, The Netherlands; ⊥ Materials Simulation and Modelling, Department of Applied Physics and Science Education, Eindhoven University of Technology, 5600 MB Eindhoven, The Netherlands; # Dutch Institute For Fundamental Energy Research (DIFFER), 5612 AJ Eindhoven, The Netherlands

## Abstract

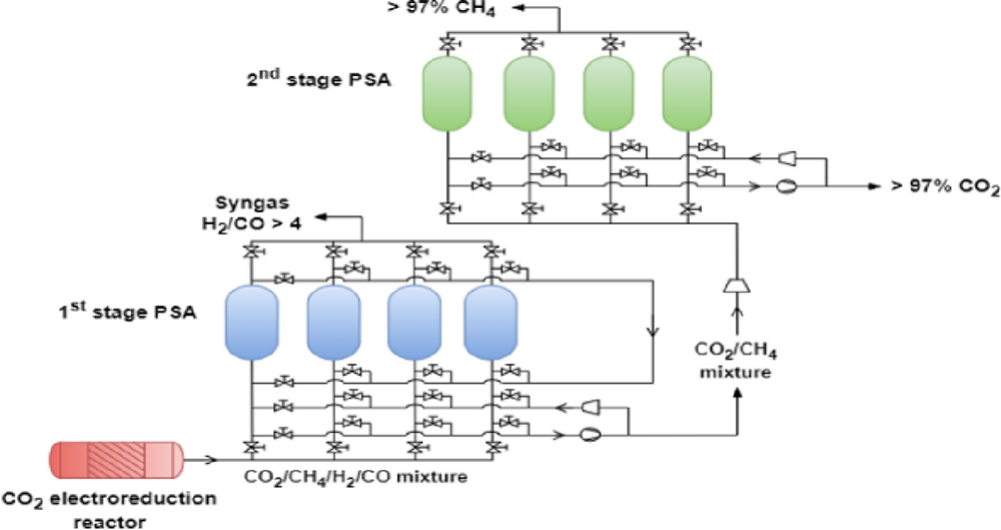

In this work, two industrial dual-step pressure swing adsorption (PSA) processes were designed and simulated to obtain high-purity methane, CO_2_, and syngas from a gas effluent of a CO_2_ electroreduction reactor using different design configurations. Among the set of zeolites that was investigated using Monte Carlo and molecular dynamics simulations, NaX and MFI were the ones selected. The dual-PSA process for case study 1 is only capable of achieving a 90.5% methane purity with a 95.2% recovery. As for case study 2, methane is obtained with a 97.5% purity and 95.3% recovery. Both case studies can produce CO_2_ with high purity and recovery (>97 and 95%, respectively) and syngas with a H_2_/CO ratio above 4. Although case study 2 allows methane to be used as domestic gas, a much higher value for its energy consumption is observed compared to case study 1 (64.9 vs 29.8 W h mol_CH4_^–1^).

## 1 Introduction

Carbon dioxide, which accounts for about 80% of the anthropogenic greenhouse gas (GHG) emissions, is the major contributor to global warming.^1^ The Kyoto Protocol (1997) was the first international climate agreement that aimed to reduce the total emissions of GHG, mainly CO_2._^2^ Nearly two decades later, 196 countries accepted, under the Paris Climate Agreement (2015), to limit the increase in the average global temperature by 2 °C.^3^ The rise in global CO_2_ emissions and the depletion of carbon-based energy resources have been predominately driven by the increasing energy consumption, especially from fossil fuels.^4,5^ Although 2020 registered the most significant decline in global CO_2_ emissions (−5.8% relative to the previous year), this was an atypical year caused by the COVID-19 outbreak.^6^

There are several approaches to reduce anthropogenic CO_2_ emissions, namely, efficiency improvement, which leads to the reduction of energy consumption; large-scale replacement of fossil fuels by renewable energy sources; and implementation of carbon capture and storage (CCS) or carbon capture and utilization (CCU) systems. Although the best way to reduce GHG emissions is using 100% renewable energy sources, this is still not technoeconomically feasible, and the supply chains are not ready. So, fossil fuels will continue to be indispensable for fulfilling society’s energy needs in the short to medium terms.^7,8^ Thus, CCS emerges as a promising method toward energy decarbonization, involving carbon capture from large CO_2_ point-emission units, namely, fossil-fuel-based power plants and energy-intensive industries like oil refineries and cement production, which is followed by its transport and permanent storage in deep oceans or geological formations (*e.g.*, underground saline aquifers).^8−10^ The downsides of CO_2_ storage include being expensive and wasting the potential utilization of captured CO_2_ in various industrial applications. Besides, it is not sure that the stored CO_2_ would remain entirely contained over the years.^9^ More recently, CCU has gained the attention of many research organizations and industrial counterparts because it suggests an alternative to CO_2_ sequestration. In this approach, the captured CO_2_ is utilized as a commodity or feedstock in fuel/chemical synthesis processes, creating a revenue stream for the recovered CO_2_ and improving the economic feasibility of carbon capture systems.^8^

The utilization of captured CO_2_ can be divided into two categories: CO_2_ physical utilization and CO_2_ conversion into value-added chemicals/fuels.^8,9^ In physical utilization, CO_2_ molecules remain pure or are dissolved in a mixture. This compound can be directly used in carbonated beverages and fire extinguishers or as dry ice, solvent, refrigerant, and process fluid. However, these applications are limited in scale and have a negligible effect on reducing CO_2_ emissions.^8^ Another CO_2_ utilization route is to improve large-scale processes such as enhanced oil recovery (EOR), enhanced gas recovery (EGR), and enhanced geothermal systems (EGSs).^1,8^ Alternatively, carbon dioxide can be employed as a raw material in chemical or fuel synthesis processes, where its molecular bonds are broken and converted into numerous products, such as CO, syngas (a mixture mainly composed of H_2_ and CO), methane, methanol, formic acid (HCOOH), and formaldehyde (HCHO).^8^ The main challenge of CO_2_ conversion technologies is overcoming the high stability of the CO_2_ molecules because the split of their double bonds (O=C=O) requires a large amount of energy, which imposes limitations on their industrial-scale use.^1,9^ Moreover, fuels synthesized from recovered CO_2_ face a further issue because they can be two or three times more costly than conventional fossil fuel competitors. Thus, extensive research on large-scale CO_2_ recycling and conversion into synthetic fuels is needed to improve their economic viability.^11^ In particular, CO_2_ electroreduction processes have been broadly investigated to produce value-added fuels, promoting a net-zero carbon economy, mainly if the electricity is generated from renewable energy sources.^12,13^

The CO_2_ electrocatalytic reduction, which is generally conducted in an H-type or flow cell reactor,^1,14^ involves initial CO_2_ chemical adsorption and activation to form CO_2_^·^^–^ on the active sites of the catalyst surface followed by successive electroreduction/protonation steps through the transfer of electron (e^–^)/proton (H^+^) pairs. The overall number of e^–^/H^+^ pairs transferred throughout this CO_2_ conversion process leads to different final products; for example, CO and HCOOH correspond to a transfer of two, HCHO of four, methanol of six, and methane of eight e^–^/H^+^ pairs. After the multielectron/multiproton transfer steps, the reduced product, obtained in the cathodic compartment, desorbs from the catalyst surface.^12,15^Table S1 lists the standard reduction potentials of the reduction half-reactions vs the normal hydrogen electrode (NHE) at a pH of 7, and Figure S1 presents a scheme of the CO_2_ electroreduction reaction.

Among the possible reduced products, methane synthesized via CO_2_ electroreduction has attracted significant interest because it represents a carbon-neutral alternative to natural gas and thus a sustainable route to overcome fossil fuel dependence.^14,16^ Although the CO_2_ reduction to methane presents a more positive standard reduction potential than other CO_2_ reduction reactions, which is thermodynamically more favorable, it also requires more electrons, and thus, it has slow kinetics. Consequently, this reaction is usually accompanied by the formation of other products, particularly those with fewer electron transfers, such as CO and HCOOH, which makes obtaining a high CH_4_ selectivity more challenging.^17^ Additionally, the hydrogen evolution reaction (HER) derived from water reduction, which has a relatively positive thermodynamic potential and involves a two-electron transfer, may also compete with the CO_2_ reduction to CH_4_ in aqueous electrolytes, forming H_2_ as the major secondary product in the cathodic compartment. Hence, the design of efficient electrocatalysts is crucial to inhibiting HER and promoting the desirable CO_2_ reduction reaction.^15,18^ Copper-based catalysts are the ones that show more capacity in electrocatalytic reduction of CO_2_ into hydrocarbons with good Faradaic efficiencies despite the current difficulty in directly obtaining C1 products such as CH_4_ with sufficient selectivity to be employed in practical applications.^14,17,19,20^ So, to produce highly pure methane, the CO_2_ electrochemical reduction subproducts, namely, H_2_, CO, and unconverted CO_2_, can be posteriorly removed by a separation unit, particularly a pressure swing adsorption (PSA) process.^16,21,22^

This work focused on developing a dual-PSA technology capable of producing a high-purity methane stream and simultaneously recovering CO_2_ and syngas from a gas effluent of a CO_2_ electroreduction reactor. The gas composition strongly depends on the experimental conditions, *i.e.,* gas inlet flow rate, imposed potential, CO_2_ crossover via the membrane, and nature and morphological characteristics of the catalyst.^23^ Thus, there is no unique outlet gas composition of the CO_2_ electroreduction reactor; however, as a case study, we chose to examine a gas composition of 40%_v_ of CO_2_, 27%_v_ of CH_4_, 27%_v_ of H_2_, and 6%_v_ of CO, which is representative of the literature and imposes a challenge of separation.^19^ Methane is widely utilized as a fuel for cooking, domestic heating, and transport, as well as a feedstock and/or energy source in power plants and industries.^9,24^ To be used as domestic gas, methane must have a purity above 97%.^25^ Alternatively, methane can be directly injected into natural gas grids; the required methane specifications for some countries can be found in Awe et al.^26^ and Kapoor et al.^27^ For instance, in France, methane must have a purity equal to or above 86%_v_ with CO_2_, H_2_, and CO contents below 2.5, 6, and 2%_v_, respectively, to be injected in natural gas grids.^26,27^ Furthermore, the unconverted CO_2_ should be recovered and recycled back into the electroreduction unit to maximize the CO_2_ conversion,^16^ whereas the obtained syngas stream could be further utilized, for instance, in the Fischer–Tropsch process for the synthesis of higher-value fuels, preferably with a H_2_/CO ratio of around 2.^28^

The PSA-based separation is a reliable technology for methane purification due to its high energy efficiency, simplicity, low investment and operating costs, and limited footprint.^22,25^ Activated carbon, metal–organic frameworks, carbon molecular sieves, and zeolites are some available adsorbents used in CO_2_/CH_4_/syngas separation processes.^25,29,30^ Zeolites, which are porous crystalline aluminosilicates with frameworks made of SiO_4_ and AlO_4_ tetrahedra linked through shared oxygen atoms,^31^ present numerous advantages, for instance, high thermal stability, high surface area, low cost, and simple ion exchange, particularly the synthetic ones such as A, X, ZSM-5, beta, and Y.^32^

To achieve the objective of this study, the screening of suitable zeolites for the CH_4_/CO_2_/syngas separation was done in the first place. The adsorption isotherms of pure and multicomponent mixtures in the selected zeolites were then obtained through molecular simulations. Last, PSA simulations were performed to separate CH_4_, CO_2_, and syngas using different design configurations. The novelty of this work is the combination of molecular simulation screening with PSA simulations to treat an electroreduction reactor exhaust gas using zeolites. So, a multiscale modeling framework is presented here from atomistic to industrial plant scale. Furthermore, the display of adsorption equilibrium data for a mixture of CO_2_, CH_4_, H_2_, and CO on the NaX and MFI zeolites at a temperature range of 308–373 K and up to 100 bar using Monte Carlo simulations followed by a comparison of the performance between two alternative designs for a dual-PSA process capable of fractioning an electroreduction reactor outlet composed of 40%_v_ of CO_2_, 27%_v_ of CH_4_, 27%_v_ of H_2_, and 6%_v_ of CO into valuable products with these two zeolites is also reported for the first time.

## 2 Methods

### 2.1 Molecular Simulation Methodology

Molecular simulations were carried out to obtain adsorption equilibrium properties of a gas mixture resulting from the CO_2_ electroreduction reaction, where the initial composition is 40%_v_ of CO_2_, 27%_v_ of CH_4_, 27%_v_ of H_2_, and 6%_v_ of CO. Adsorption isotherms were obtained for pure compounds as well as multicomponent mixtures using Monte Carlo simulations in the grand canonical ensemble (GCMC). In this open ensemble, the pressure and fugacity are related via the fugacity coefficient, and the chemical potential is obtained from the Peng–Robinson equation of state.^33^ The adsorption isotherms are obtained by averaging 10^5^ MC cycles for a single component and 5 × 10^5^ cycles for mixtures after 10^4^ MC equilibration cycles. All the simulations are performed using the molecular simulation package RASPA.^34,35^ The models used for carbon dioxide and carbon monoxide are reported in Harris and Yung^36^ and Martin-Calvo et al.,^37,38^ respectively. These two models consist of van der Waals interaction centers modeled with 12-6 Lennard–Jones potential and point charges. Methane^39^ and hydrogen^40,41^ are modeled as noncharged spherical molecules with an effective interaction center. The Lennard–Jones parameters to define host–guest interactions were taken from the literature.^40,42−44^ In addition, molecular dynamics (MD) simulations were performed in the NVT ensemble to calculate the mean square displacement to assess the diffusion of the adsorbates at 373 K and at infinite dilution, *i.e.*, a single molecule confined within the zeolite pores. The temperature was fixed with the Nosé–Hoover thermostat,^45,46^ and the time step was set to 1 fs.

During the simulations, the zeolites are modeled as rigid frameworks with oxygen, silicon, and aluminum atoms at crystallographic positions, whereas sodium cations are allowed to move. The relaxed structures of the zeolites, their configurations, and the initial position of the cations were taken from our previous works.^47,48^ The pore size distribution (PSD) of the materials is computed geometrically as described in the literature.^49,50^Figure S2 depicts the structure and pore size distribution of the selected adsorbents. An initial set of nine zeolites was chosen to cover a variety of topologies, pore sizes, and chemical compositions. ATV, MTW, and AFI are one-dimensional channels like zeolites with different pore sizes. LTA and FAU zeolites contain big interconnected cavities. Regarding LTA topology, the pure silica ITQ-29 zeolite was used, whereas for FAU, three zeolites with varying Si/Al contents were used. High-silica HS-FAU is a highly dealuminated zeolite (Si/Al ≈ 100), and NaY (Si/Al = 2.56) and NaX (Si/Al = 1.06) have a noticeable amount of sodium extraframework cations, which have a strong influence on the adsorption behavior. MFI is a well-known commercial zeolite with interconnected channels with pore and window sizes slightly bigger than the size of the adsorbates. Finally, the BRE zeolite was used and proposed as a good candidate for the separation of small gases, including CO and CO_2_.^5^

The adsorption equilibrium data of CH_4_, CO_2_, CO, H_2_, and respective mixtures on the selected adsorbents (NaX and MFI zeolites) are well described by the dual-site Langmuir (DSL) model, which assumes that the adsorbent surface is heterogeneous and considers two different types of adsorption sites:^51^
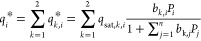
1
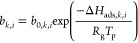
2where *i* is each component, *q*_i_^*^ is the adsorption equilibrium concentration of component *i*, *k* is each type of adsorption site, *q*_*k*, i_^*^ is the adsorption equilibrium concentration of component *i* on adsorption site *k*, *q*_sat, *k*, *i*_ is the saturation capacity for component *i* on adsorption site *k*, *n* is the total number of components, *b*_*k*, *i*_ is the affinity constant of component *i* on the adsorption site *k*, *b*_0, *k*, *i*_ is the affinity constant of component *i* on adsorption site *k* at infinite temperature, −Δ*H*_ads, *k*, *i*_ is the isosteric heat of adsorption of component *i* on adsorption site *k*, *R*_g_ is the ideal gas constant, *T*_p_ is the solid temperature, and *P_i_* is the partial pressure of component *i*.

### 2.2 PSA Modeling

PSA units operate with multiple packed beds that alternate between two main steps: adsorption, where the adsorbent preferentially retains the heavier components contained in the gas mixture, and regeneration or desorption, where those same species are removed from the adsorbent by reducing the total bed pressure. During the adsorption step, a light product or raffinate stream mainly composed of gases with less affinity to the adsorbent is obtained. In contrast, a heavy product or extract stream mostly composed of gases with more affinity to the adsorbent is recovered in the regeneration step. It is possible to obtain valuable products from the adsorption, desorption, or both steps. The basic PSA configuration is the Skarstrom cycle composed of the following steps: pressurization with feed, feed, counter-current blowdown, and counter-current purge with the light product. The pressurization step can be made in a co-current way with the feed stream or in a counter-current way with the light product. Then, in the feed step, the light product is obtained at high pressure, which is followed by the blowdown and purge steps, where the column is regenerated, generating the heavy product at low pressure.^52^ Pressure equalization and rinse steps are commonly added in cyclic adsorptive processes to decrease the energy consumption of the system and/or to improve the product recovery. The rinse step consists of passing the heavy product through the bed before blowdown^25^ and contributes to improve the purity of the heavy product.

The dynamic behavior of adsorption in a packed bed is represented through a mathematical model that must include mass, energy, and momentum balances. The development of the mathematical model to be employed in this study is based on the following assumptions: ideal gas behavior; axial dispersed plug flow; external mass and heat transfer resistances expressed with the film model; particle mass transfer resistance expressed with the linear driving force (LDF) model; no temperature gradients inside each particle, as the heat transfer in the solid phase is much faster than in the gas phase; the column wall interchanges energy with the gas phase inside the column and with the external environment; constant porosity along the bed; and the Ergun equation is valid locally.^53^ The Ergun equation is commonly used for gas flow through packed columns, where the superficial velocity term changes throughout the bed for compressible fluids. For significant pressure drops, this correlation should be applied locally by expressing the pressure gradient in the differential form.^54^ Although dispersion can occur in two directions within the bed, *i.e.*, radial and axial, when the adsorbed bed diameter is much larger than the particle diameter, the radial dispersion is considered insignificant, and the radial concentration of the gas phase is assumed to be uniform.^52^ The mass, energy, and momentum balance equations of the mathematical model of an adsorption fixed bed are presented in Table 1.

**Table 1:** Mass, Energy, and Momentum Balance Equations of the Mathematical Model of an Adsorption Fixed Bed

mass balances	
gas phase	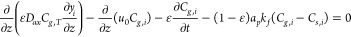
solid phase	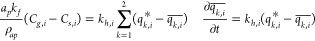

Energy Balances	
gas phase	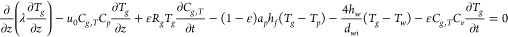
solid phase	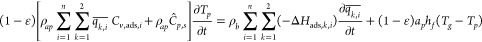
column wall	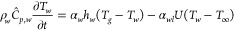 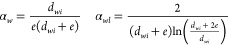

Momentum Balance	
Ergun Equation	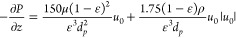

Transport and thermodynamic parameters must be defined to complete the mathematical model. Thereby, the Wakao and Funazkri correlations are used to calculate the axial mass and heat dispersion coefficients and the mass and heat transfer coefficients,^52,55^ whereas the molecular diffusivity is obtained by the Chapman–Enskog equation.^56^ The convective heat transfer coefficient between the gas and the wall is calculated with the Wasch and Froment correlation.^55^ General properties of the gases like density, viscosity, thermal conductivity, and molar specific heat were obtained according to Prausnitz et al.^56^ The molar specific heat of the adsorbed gas is assumed to be equal to the one in the gas phase.^55^

The corresponding boundary conditions for each step of the PSA cycle are given in Table 2. The pressure is controlled at the outlet (*P*_exit_); indeed, the pressure during equalization–depressurization and blowdown steps is considered to decrease exponentially with time to represent the behavior of a valve.

**Table 2:** Boundary Conditions for Each Step of the PSA Cycle

	*z* = 0	*z* = L
counter-current pressurization with light product		
	*u*_0_ = 0	*u*_0inlet_*C*_inlet, *T*_ = *u*_0_*C*_*g*, *T*_
	
feed/rinse		
	*u*_0inlet_*C*_inlet, *T*_ = *u*_0_*C*_*g*, *T*_	*P* = *P*_exit_
	
pressure equalization–depressurization		
	*u*_0_ = 0	*P* = *P*_exit_
	
blowdown		
	*P* = *P*_exit_	*u*_0_ = 0
	
purge		
	*P* = *P*_exit_	*u*_0inlet_*C*_inlet, *T*_ = *u*_0_*C*_*g*, *T*_
	
pressure equalization–pressurization		
	*u*_0inlet_*C*_inlet, *T*_ = *u*_0_*C*_*g*, *T*_	*u*_0_ = 0
	

The mathematical model was implemented in gPROMs ModelBuilder and numerically solved using the orthogonal collocation on finite elements method (OCFEM) with second-order polynomials and 40 intervals. The cyclic simulations were carried out until a cyclic steady state (CCS) was attained.

## 3 Results and Discussion

### 3.1 Adsorption Equilibrium

First, the study of the adsorption equilibrium of the four adsorbates of the targeted mixture in the nine initial zeolites was carried out. Figure S3 shows the pure component adsorption isotherms of CO_2_, CH_4_, CO, and H_2_ in all the zeolites at 308 K. This was the temperature selected to do a first adsorbent screening. The preferred component in most zeolites is CO_2_, except for ATV and BRE, which adsorb more CH_4_ and CO than CO_2_. This unexpected behavior can be due to the better fitting of these two gases with the narrow pores of these two zeolites. CH_4_ and CO have a similar kinetic diameter of about 3.8 Å, which is close to the size of the ATV (4.2 Å) and BRE (4.7 Å) cavities. All zeolites generally adsorb slightly more CH_4_ than CO except NaY and NaX, which show no significant preference over any of the two molecules. Finally, because of the small size of the H_2_ molecule, it is the less adsorbed component in this set of zeolites.

To analyze the competitive adsorption of these gases, Figure S4 collects the adsorption isotherm of the quaternary mixture (CO_2_: 0.40; CH_4_: 0.27; H_2_: 0.27; CO: 0.06). As in the pure component isotherms, CO_2_ is the most adsorbed gas except in ATV and BRE zeolites. It is possible to observe how NaY and NaX zeolites mostly adsorb CO_2_ and almost exclude the rest of the components. In an ideal situation, all CO_2_ could be removed in a first step of a separation process, resulting in a ternary mixture (CH_4_: 0.45; H_2_: 0.45; CO: 0.1). The results of this ternary mixture are shown in Figure S5. Once CO_2_ is removed from the mixture, all the zeolites follow the same trend, *i.e.*, CH_4_ > CO > H_2_. This work aims to obtain methane and CO_2_ with high purity and maximize their recovery. To compare the performance of each zeolite for the separation of the components of the quaternary and ternary mixtures, the adsorption selectivity (*S*_ads_) was computed in a multicomponent mixture, defined as:
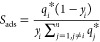
3where *y_i_* is the molar fraction of component *i*, which is the component of interest in each case. Figure S6 collects the adsorption selectivities of the multicomponent mixtures in the 1–10 bar pressure range. It was found that NaY and NaX outperform the rest of the zeolites to efficiently separate carbon dioxide from the quaternary mixture. However, these two zeolites are the less suitable adsorbents for separating methane from the remaining ternary mixture. On the contrary, ATV, BRE, MTW, and MFI show a more promising separation factor in this case with similar adsorption selectivity in favor of methane. However, because of the narrow pores of ATV and BRE, these two zeolites could show diffusion limitations that hinder their adsorption equilibrium performance. Additional MD simulations were performed with a single molecule in each zeolite to gain insight into the diffusion of the adsorbates in confinement. Figure S7 shows the mean squared displacement (MSD) of each gas at infinite dilution in all studied zeolites. The BRE zeolite shows a flat MSD that does not reach a clear diffusive regime like the other zeolites. In addition, ATV also reveals diffusion limitations for methane. These results are consistent with the pore and window sizes where the molecules can diffuse (Table S2). Thus, ATV and BRE were discarded as possible candidates for the targeted separation.

**Figure 1:**
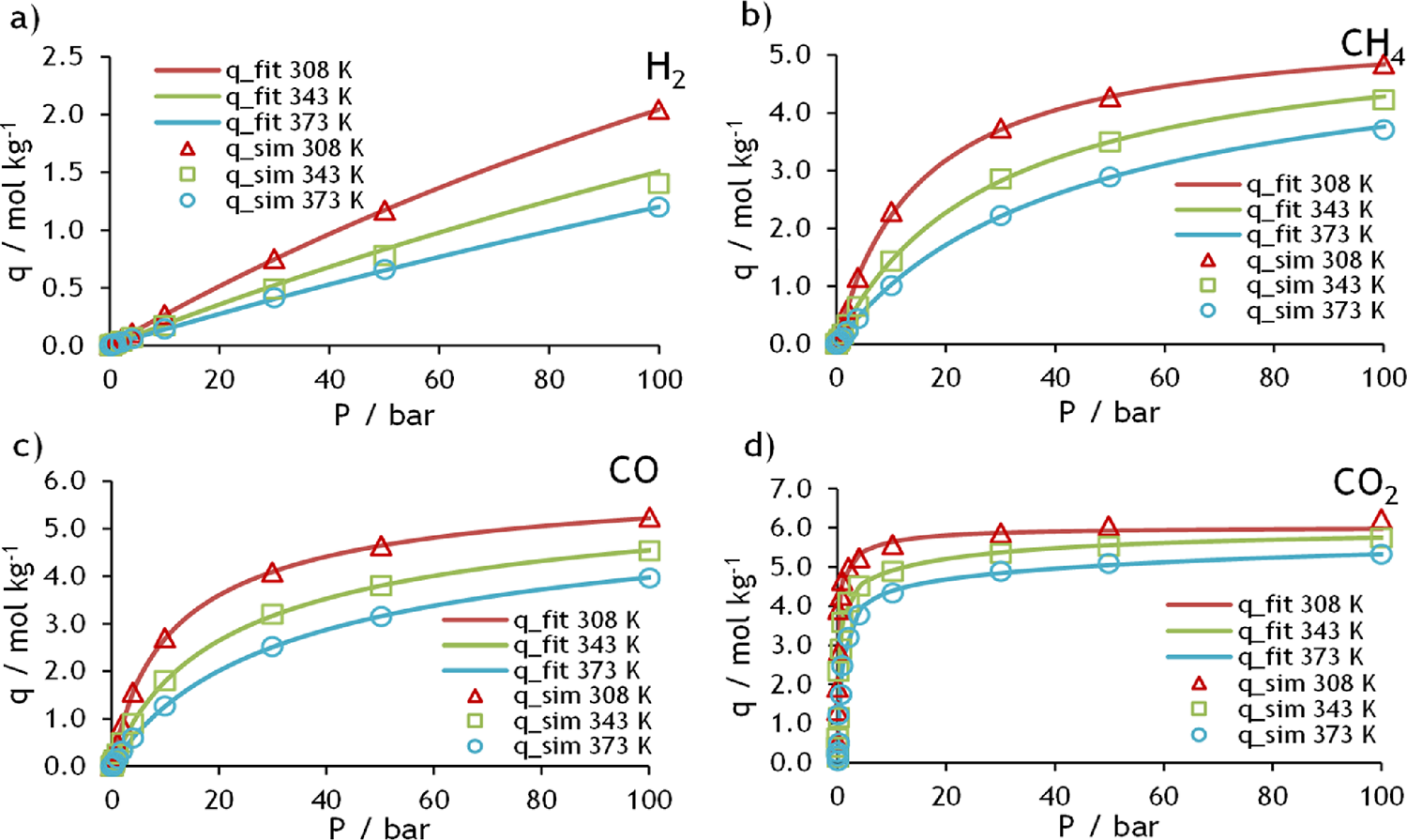
Adsorption equilibrium isotherms on NaX for (a) H_2_, (b) CH_4_, (c) CO, and (d) CO_2_ at 308, 343, and 373 K and up to 100 bar.

**Figure 2:**
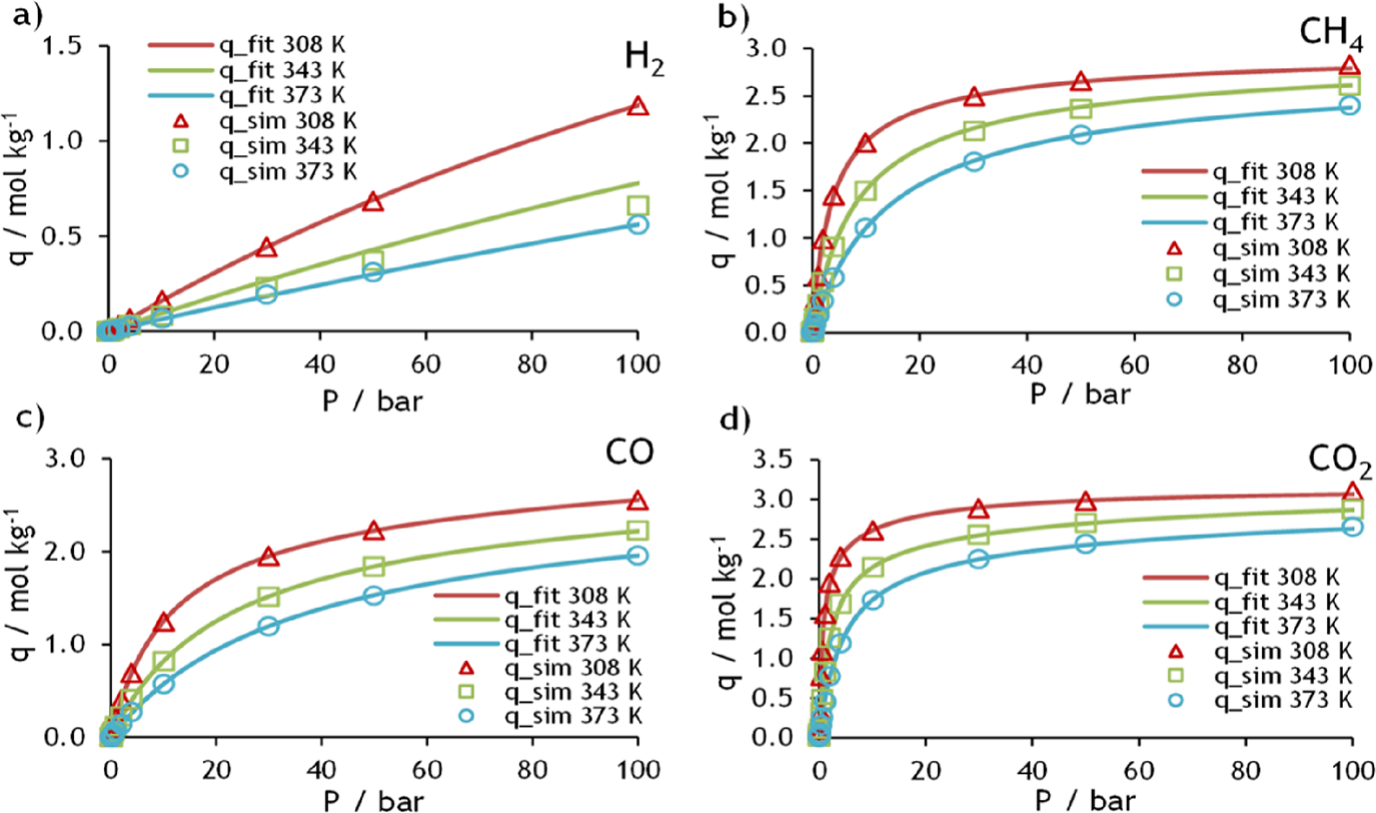
Adsorption equilibrium isotherms on MFI for (a) H_2_, (b) CH_4_, (c) CO, and (d) CO_2_ at 308, 343, and 373 K and up to 100 bar.

On the basis of the previous results, NaY and NaX zeolites were proposed for the removal of CO_2_ from the initial quaternary mixture and MTW and MFI zeolites to obtain high-purity methane from the secondary ternary mixture. However, because of the commercial availability of NaX and MFI, these two zeolites were the ones selected for designing and analyzing in detail a PSA separation process. For this purpose, it is essential to fit the single and multicomponent adsorption values to a suitable isotherm model. The adsorption equilibrium data on NaX and MFI zeolites are presented in Figures 1 and 2, respectively. In these figures, symbols represent the molecular simulation data, and solid lines represent the DSL isotherm fit considering pure component data and multicomponent equilibrium data.

The DSL parameters for both adsorbents can be found in Table 3.

**Table 3:** DSL Parameters for CH_4_, CO_2_, CO, and H_2_ Using NaX and MFI Zeolites

	*b*_O,1_ (bar^–1^)	( – Δ*H*)_1_ (kJ mol^–1^)	*q*_sat,1_ (mol kg^–1^)	*b*_0,2_ (bar^–1^)	( – Δ*H*)_2_ (kJ mol^–1^)	*q*_sat,2_ (mol kg^–1^)
NaX						
H_2_	9.8 × 10^–5^	9.3	6.5	1.5 × 10^–5^	12.9	1.7
CH_4_	3.0 × 10^–4^	13.9	4.3	2.0 × 10^–6^	26.2	1.3
CO	1.6 × 10^–4^	17.1	4.2	3.3 × 10^–6^	22.6	1.9
CO_2_	9.7 × 10^–6^	36.3	4.6	2.3 × 10^–9^	48.0	1.4
MFI						
H_2_	6.1 × 10^–7^	14.0	3.6	1.9 × 10^–5^	13.8	3.9
CH_4_	7.4 × 10^–6^	21.2	0.4	9.3 × 10^–5^	20.7	2.6
CO	1.6 × 10^–6^	18.9	1.4	1.1 × 10^–4^	17.2	2.5
CO_2_	6.8 × 10^–8^	35.9	0.7	2.7 × 10^–5^	28.1	2.4

**Figure 3:**
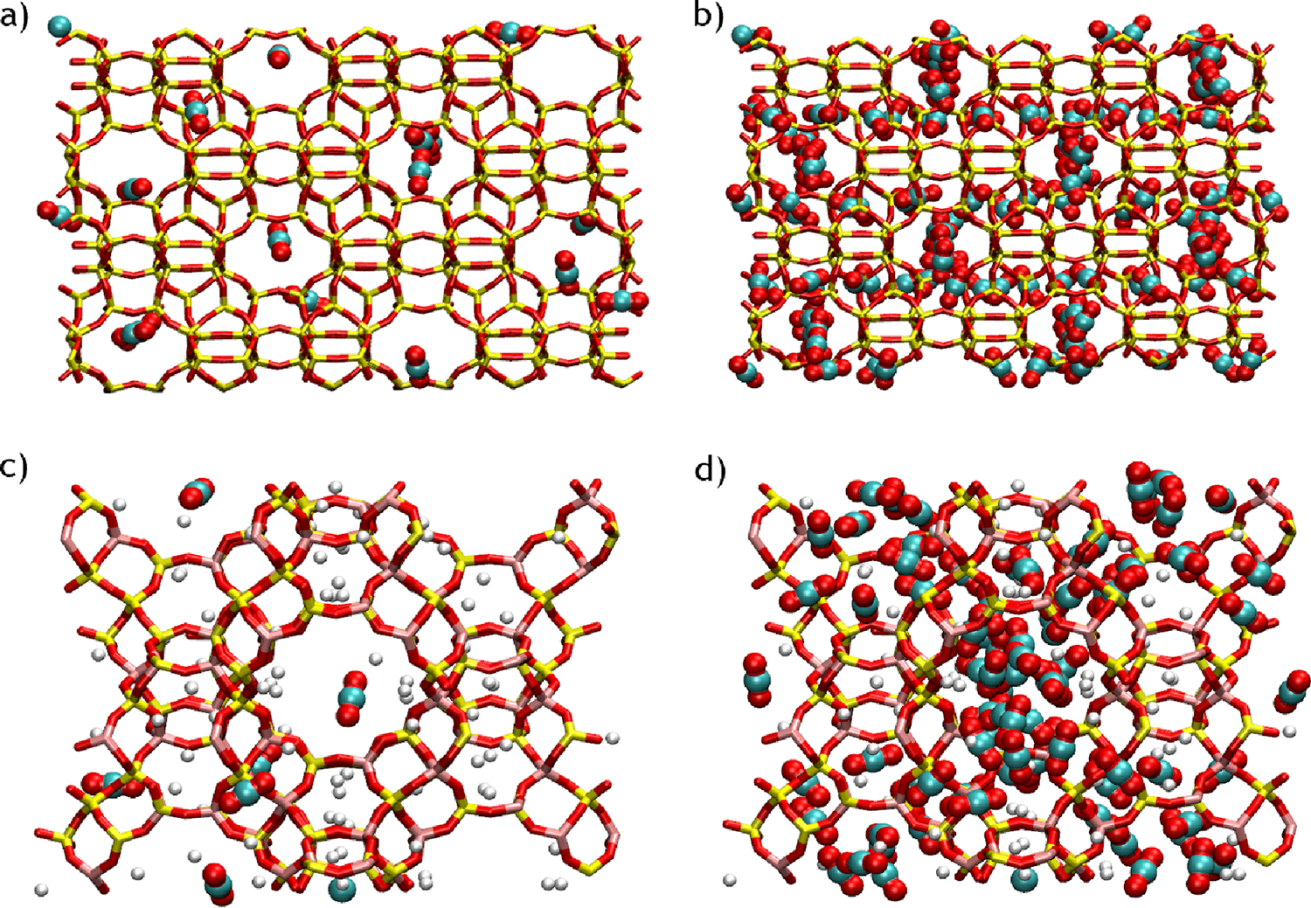
Snapshots of the adsorption equilibrium of CO_2_ on the MFI zeolite (a) at low loadings and (b) high loadings and on the NaX zeolite (c) at low loadings and (d) high loadings.

As previously mentioned, the DSL model accounts for the heterogeneity of sites. To better understand and visualize the differences in adsorption equilibrium in the two distinct sites of each zeolite, snapshots of the adsorption equilibrium of CO_2_ at low loadings and high loadings for each zeolite are shown in Figure 3. The MFI zeolite presents a structure with intersecting channels,^57^ with the adsorption equilibrium of the components on the channels (straight and zigzag) being different from the one on the intersections. As can be seen in the snapshots, CO_2_ molecules preferentially fill the intersections before occupying the straight and zigzag channels, in concordance with previous observations on the adsorption of hydrocarbons in MFI.^58^ On the other hand, the NaX zeolite presents a structure that contains large cavities and sodalite cages,^57,59^ and so it also contains two distinct sites. However, the sodalites are not an adsorption site for CO_2_, CO, and CH_4_; only H_2_ can fit in them. In addition to these cavities, NaX contains a high concentration of sodium cations that can act as heterogeneous adsorption sites because of their interaction with the guest molecules. Therefore, there is no clear indication of the presence of different adsorption sites for the NaX zeolite for all the components (see Figure 3), but the DSL model proved to predict well the molecular simulation data obtained in this work and is widely used in the fitting of adsorption equilibrium data on zeolites. Moreover, Figure S8 shows the adsorption equilibrium data and corresponding DSL fit for CO_2_, CO, CH_4_, and H_2_ as pure components on the other analyzed zeolites (ATV, BRE, MTW, AFI, ITQ-29, HS-FAU, NaY) at 308 K and up to 100 bar, whereas Table S3 presents the DSL parameters for these zeolites.

The properties of the adsorbents used in the PSA simulations are shown in Table 4.

**Table 4:** Properties of the NaX and MFI Zeolites

adsorbent	NaX^60,61^	MFI^62^
particle diameter (m)	0.001	0.0014
particle density (kg m^–3^)	690	1070
solid heat capacity (J kg^–1^ K^–1^)	900	1000

### 3.2 PSA Simulations

**Figure 4:**
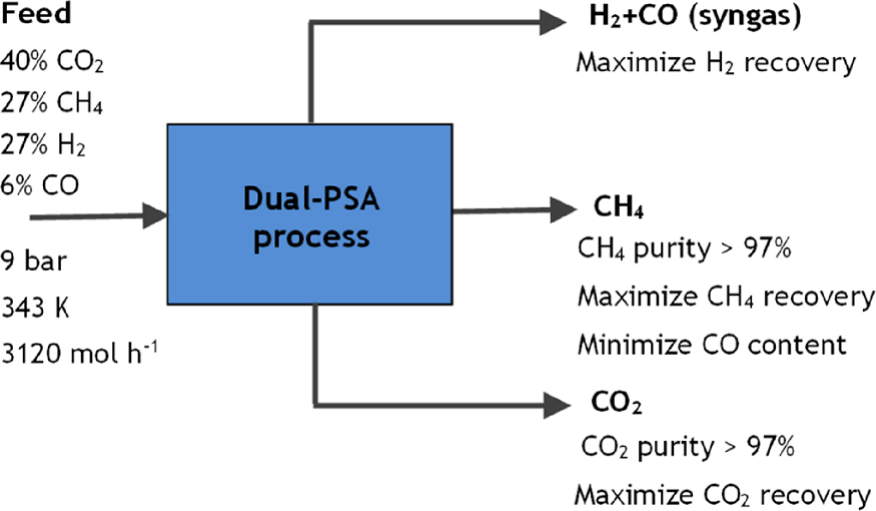
Block diagram with the overall process specifications.

In this work, it is intended to develop an industrial dual-PSA process capable of producing methane with high purity from a feed mixture with a molar flow rate of 3120 mol h^–1^ and composed of 40% of CO_2_, 27% of CH_4_, 27% of H_2_, and 6% of CO at 343 K and 9 bar while also considering the valorization of carbon dioxide and syngas. A temperature of 343 K was chosen for the PSA simulations because it allows a better adsorbent regeneration than lower temperatures (such as 308 K) because the curvature of the adsorption equilibrium isotherm of CO_2_ is less sharp at higher temperatures for a low-pressure range. Figure 4 shows the overall process specifications. The main objective of this study is to obtain methane and carbon dioxide with a purity above 97%, maximizing their recoveries.

**Figure 5:**
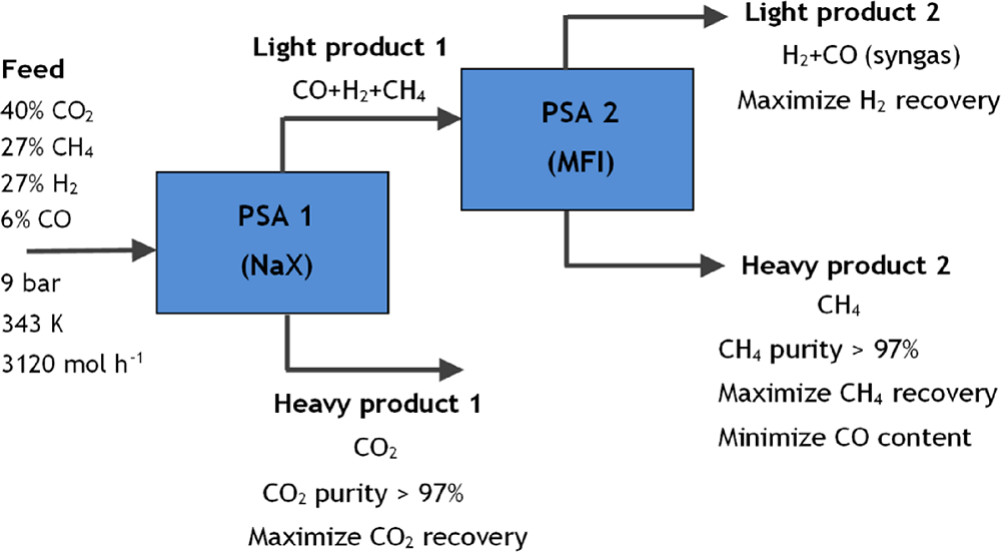
Block diagram with process specifications for case study 1.

The dual-PSA process was designed and simulated using two alternative configurations, with the main difference between them being the order in which the separation of CO_2_/CH_4_/syngas occurs. In the first configuration (case study 1), carbon dioxide is removed from the feed mixture using NaX zeolite, and subsequently, methane is separated from the syngas stream by the MFI zeolite, as illustrated in Figure 5.

**Figure 6:**
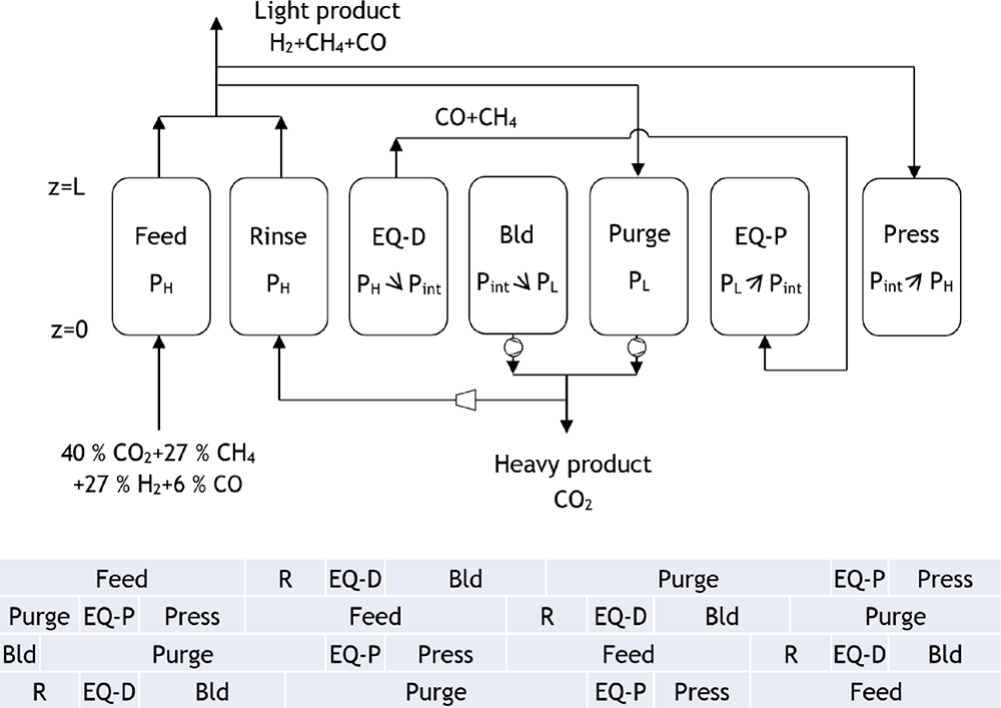
Proposed cycle for the PSA 1 of case study 1 (R: rinse, EQ-D: pressure equalization–depressurization, Bld: blowdown, EQ-P: pressure equalization–pressurization, press: pressurization).

The proposed cycle for the PSA 1 of case study 1 and the corresponding extension for a four-column unit are presented in Figure 6. This PSA cycle is composed of the following seven steps: feed, where the light product is formed at high pressure (*P*_H_); rinse, where a fraction of the heavy product passes through the column co-currently at high pressure and so more light product is generated; co-current pressure equalization–depressurization until an intermediate pressure (*P*_int_) is achieved; counter-current blowdown until low pressure (*P*_L_) is attained; purge, where a portion of the light product is fed counter-currently at low pressure, producing a heavy product at the column outlet; co-current pressure equalization–pressurization until the intermediate pressure is once again achieved; and counter-current pressurization with the light product until high pressure is reached. The extension to a four-column unit, which allows continuous feed consumption, imposes some constraints on the step times. The pressure equalization steps must have the same time period and happen simultaneously, whereas the total cycle duration needs to be four times the duration of the feed step. The feed step for this PSA unit was set to be equal to 700 s, and thus, the total cycle time is 2800 s.

The adsorption bed characteristics and the operating conditions used in the mathematical model of PSA 1 for this case study are given in Table 5. It is important to note that the initial step of the design procedure is the sizing of the adsorption columns. Because of limitations in transporting large PSA vessels, their diameter should be equal to or below 4 m.^63^ A diameter of 0.5 m was assumed for the columns of the first PSA unit of each dual-PSA process to have a reasonable PSA unit size. For a feed molar flow rate of 3120 mol h^–1^, a superficial velocity of about 0.014 m s^–1^ is obtained, within the range of typical feed superficial velocities, *i.e*., between 0.01 and 0.05 m s^–1^.^64^ The transport parameters used in the simulation of PSA 1 for this case study are shown in Table S4.

**Table 5:** Adsorption Bed Characteristics and Operating Conditions Used in the Simulations of PSA 1 for Case Study 1

bed characteristics	
bed length (m)	3
internal bed diameter (m)	0.5
bed porosity	0.37
operating conditions	
*T* (K)	343
*P*_H_ (bar)	9
*P*_L_ (bar)	0.3
*P*_int_ (bar)	4
*y*_in, feed_	CO_2_: 0.40; CH_4_: 0.27; H_2_: 0.27; CO: 0.06
*F*_in_ (mol h^–1^)	feed: 3120; rinse: 3120; purge: 10.4; press: 938
step times (s)	feed: 700; rinse: 150; EQ-D: 100; Bld: 500; purge: 800; EQ-P: 100; press: 450

**Figure 7:**
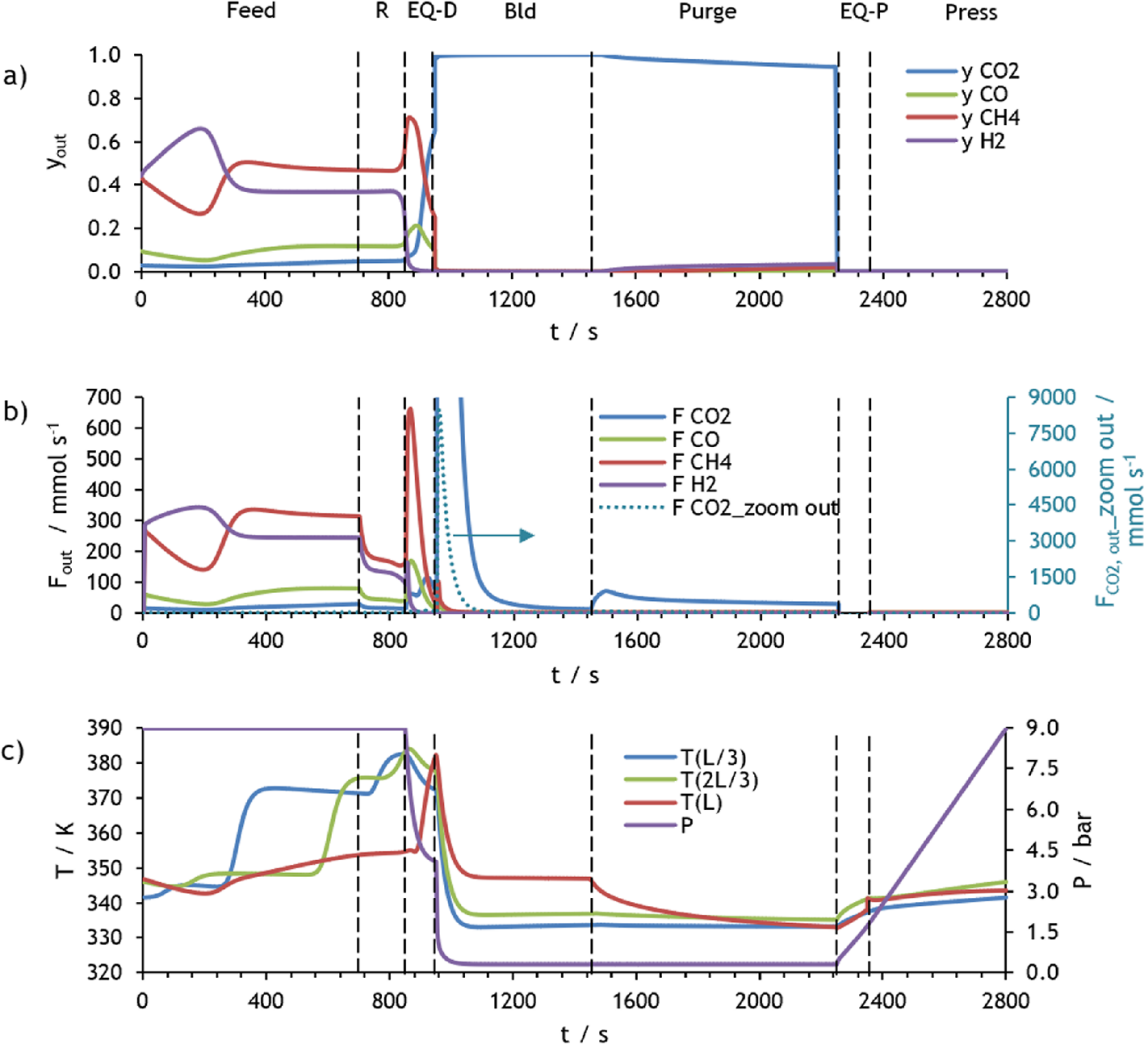
Simulation results at CSS for PSA 1 of case study 1: (a) molar fraction of each component at the column outlet, (b) molar flow rate of each component at the column outlet, and (c) pressure and temperature at three different positions within the column.

The molar fraction and molar flow rate of each component of the mixture, the pressure, and temperatures obtained during one cycle in CSS for the first PSA of case study 1 are presented in Figure 7. This figure shows that practically all carbon dioxide is adsorbed during the feed and rinse steps, which results in a rise in the bed temperature because adsorption is an exothermic process. It is crucial to notice that during rinse, a portion of the heavy product (primarily composed of CO_2_) is fed to the packed bed at high pressure to clean it, causing the lighter components to be further pushed out of the column. In the pressure equalization–depressurization time, the pressure is reduced from 9 to 4 bar, and at the end of this step, the column is filled with almost only CO_2_. The pressure is further reduced to 0.3 bar during the blowdown step, and the adsorbent is regenerated because carbon dioxide desorbs and exits the column. A decrease in the gas temperature in this step can be observed as a result of desorption being an endothermic phenomenon. The column continues to be regenerated during the purge step, in which a fraction of the light product is fed counter-currently at low pressure. Consequently, a high-purity CO_2_ product is generated during blowdown plus purge times. In the pressure equalization–pressurization and counter-current pressurization steps, the pressure increases until 1.7 and 9 bar, respectively, whereas the gas temperature slightly increases, which is related to the CO_2_ adsorption. It is possible to observe that the pressure equalization–pressurization in this PSA cycle is partial, *i.e*., the bed is pressurized from 0.3 to 1.3 bar and not until 4 bar, because further pressurization during this step compromises the purity of the desired product considerably even though an increase in the recovery continues to occur. So, in this study, a partial pressure equalization–pressurization step results in better overall process performance.

**Figure 8:**
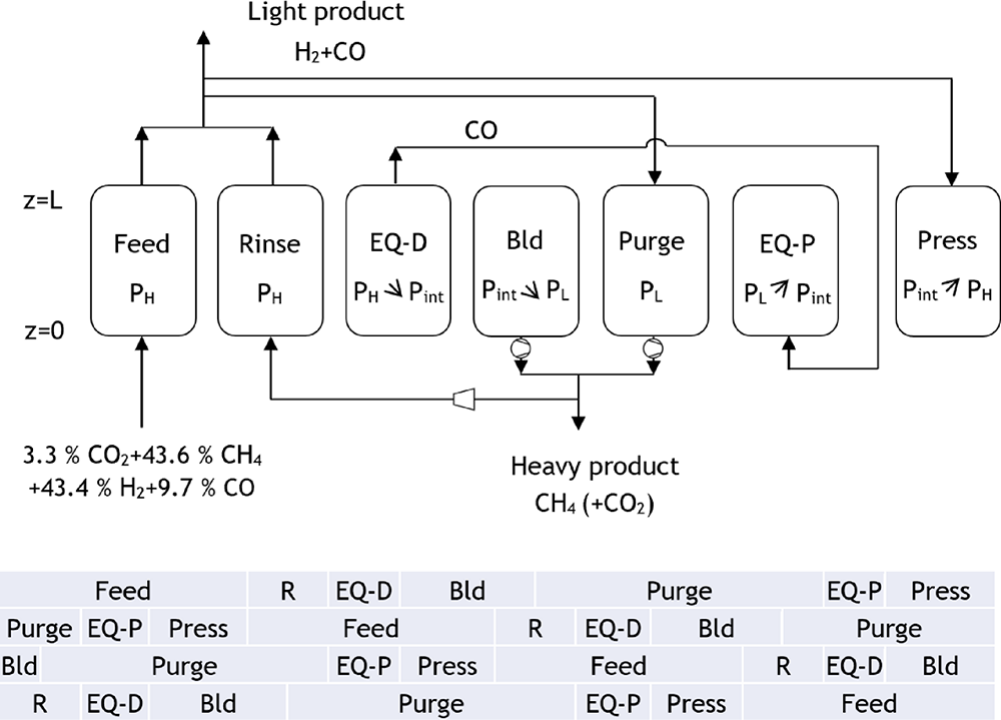
Proposed cycle for the second PSA of case study 1.

To recover methane and syngas, the light product generated in PSA 1, which contains 3.3% of CO_2_, 43.6% of CH_4_, 43.4% of H_2_, and 9.7% of CO at 343 K and 9 bar, was fed at a molar flow rate of 1923 mol h^–1^ to a second PSA in series with the first one. The proposed cycle for the PSA 2 of case study 1 and the corresponding extension for a four-column unit are displayed in Figure 8.

In Table 6, the adsorption bed characteristics and the operating conditions used in the mathematical model of PSA 2 for this case study are presented. The transport parameters used in the simulation of PSA 2 for this case study are summarized in Table S4.

**Table 6:** Adsorption Bed Characteristics and Operating Conditions Used in the Simulations of PSA 2 for Case Study 1

bed characteristics	
bed length (m)	3
internal bed diameter (m)	0.4
bed porosity	0.37
operating conditions	
*T* (K)	343
*P*_H_ (bar)	9
*P*_L_ (bar)	0.3
*P*_int_ (bar)	4
*y*_in, feed_	CO_2_: 0.033; CH_4_: 0.436; H_2_: 0.434; CO: 0.097
*F*_in_ (mol h^–1^)	feed: 1923; rinse: 1635; purge: 19.2; press: 733
step times (s)	feed: 540; rinse: 175; EQ-D: 115; Bld: 320; purge: 645; EQ-P: 115; press: 250

**Figure 9:**
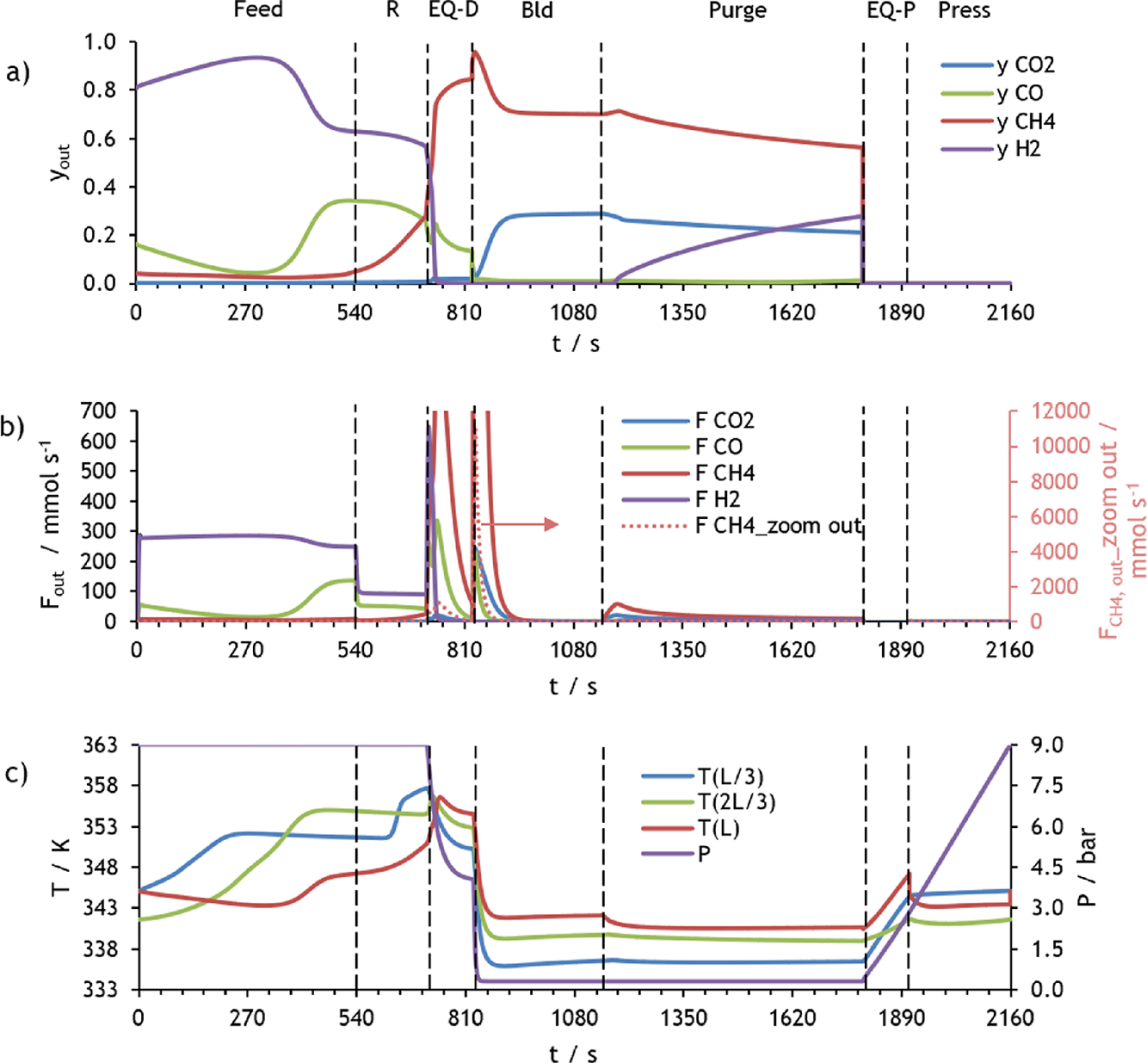
Simulation results at CSS for PSA 2 of case study 1: (a) molar fraction of each component at the column outlet, (b) molar flow rate of each component at the column outlet, and (c) pressure and temperature at three different positions within the column.

The molar fraction and molar flow rate of the components of the mixture, the pressure, and temperatures attained during one cycle in CSS for the second PSA of case study 1 are displayed in Figure 9. In this figure, it can be seen that methane is completely adsorbed in the feed step, and consequently, only syngas elutes at the column outlet. Still, it contaminates the syngas product at the end of the rinse step. At the same time, the bed temperature gradually increases. In the pressure equalization–depressurization step, the pressure is reduced from 9 to 4 bar, resulting in methane desorption and, consequently, a decrease in the bed temperature. The pressure is further reduced to 0.3 bar during the blowdown step, where CH_4_ and small amounts of CO_2_ are desorbed and exit the column. Then, a fraction of the light product (*i.e.*, syngas stream) is fed counter-currently in the purge step, which also leads to the exit of H_2_, resulting in a decrease in methane product purity. During the pressure equalization–pressurization and counter-current pressurization steps, the pressure increases to 2.9 and 9 bar, respectively, whereas the gas temperature slightly increases because of the adsorption of CH_4_ and CO_2_.

**Figure 10:**
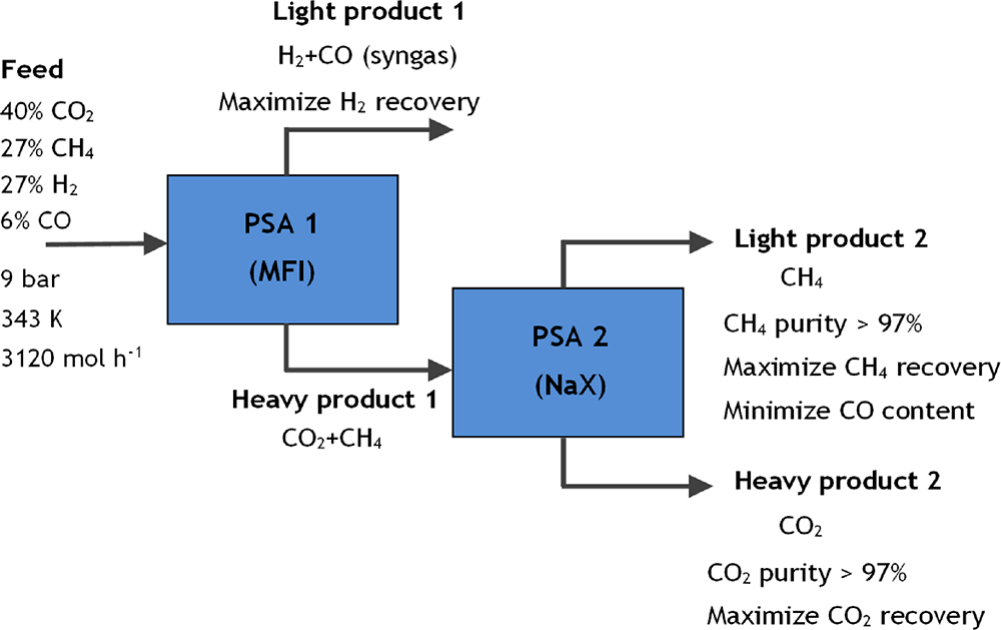
Block diagram with process specifications for case study 2.

The design of a second industrial-scale PSA process (case study 2) was made considering the process specifications presented in Figure 10. In this configuration, a mixture mostly composed of CO_2_ and methane is separated from the syngas stream with the use of the MFI zeolite. Then, CO_2_ is removed from methane using NaX zeolite.

**Figure 11:**
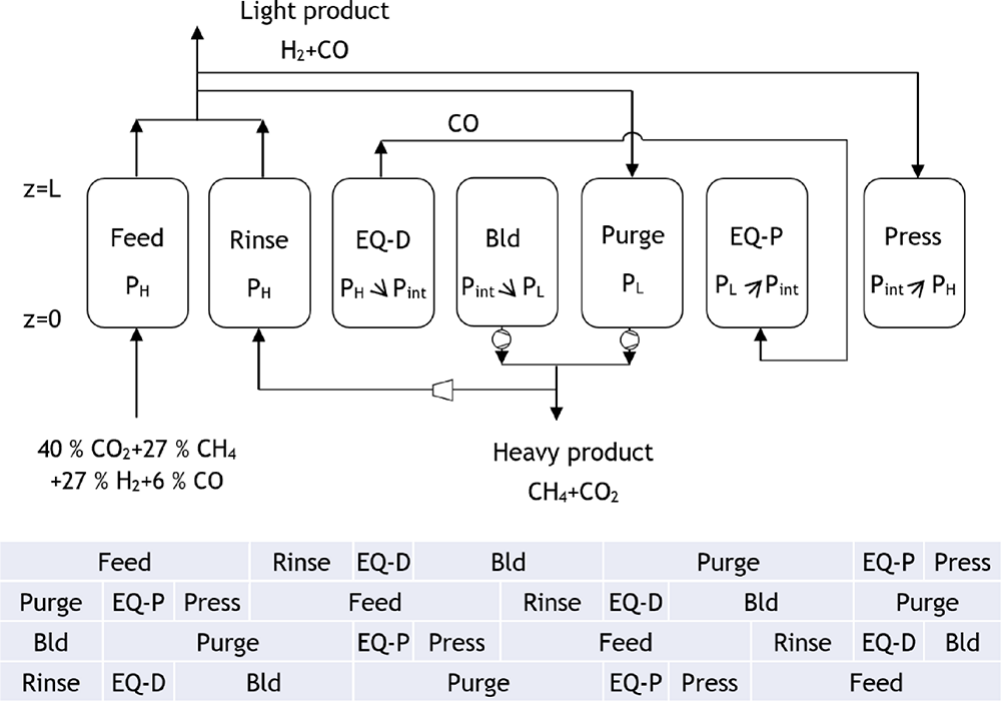
Proposed cycle for the PSA 1 of case study 2.

The proposed cycle for the PSA 1 from case study 2 and the corresponding extension for a four-column unit are presented in Figure 11. The adsorption bed characteristics and the operating conditions used in the mathematical model of PSA 1 for this case study are shown in Table 7. The transport parameters used in the simulation of this PSA are given in Table S5.

**Table 7:** Adsorption Bed Characteristics and Operating Conditions Used in the Simulations of PSA 1 for Case Study 2

bed characteristics	
bed length (m)	3
internal bed diameter (m)	0.5
bed porosity	0.37
operating conditions	
*T* (K)	343
*P*_H_ (bar)	9
*P*_L_ (bar)	0.3
*P*_int_ (bar)	4.4
*y*_in, feed_	CO_2_: 0.40; CH_4_: 0.27; H_2_: 0.27; CO: 0.06
*F*_in_ (mol h^–1^)	feed: 3120; rinse: 2964; purge: 20.8; press: 2043
step times (s)	feed: 435; rinse: 175; EQ-D: 100; Bld: 350; purge: 420; EQ-P: 100; press: 160

**Figure 12:**
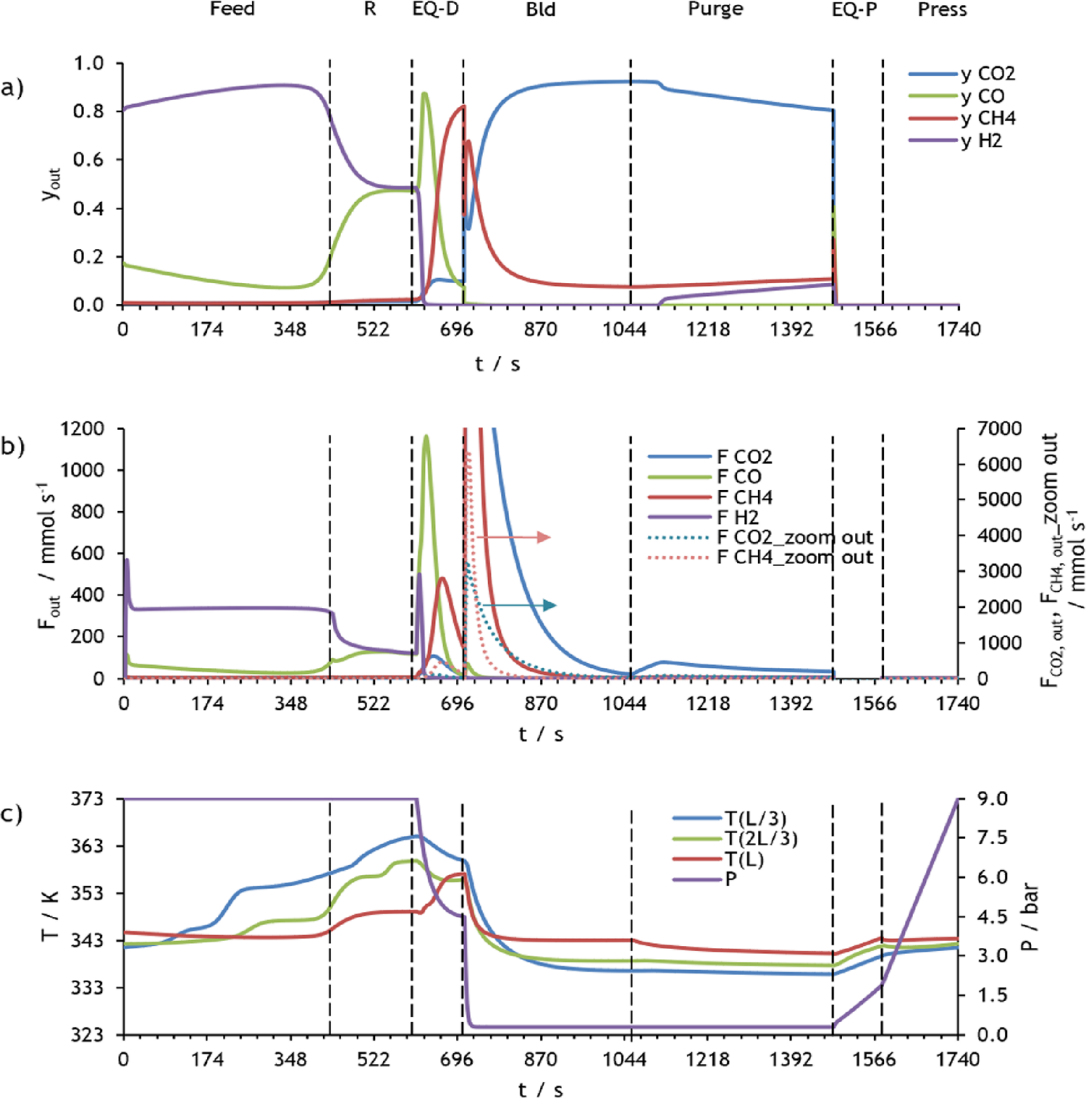
Simulation results at CSS for PSA 1 of case study 2: (a) molar fraction of each component at the column outlet, (b) molar flow rate of each component at the column outlet, and (c) pressure and temperature at three different positions within the column.

In Figure 12, the molar fraction and molar flow rate of each component of the mixture, as well as pressure and temperature values obtained during one cycle in CSS for the first PSA of case study 2, are presented.

This figure shows that during the feed and rinse steps, all carbon dioxide and methane are practically adsorbed, so these gases do not contaminate the syngas product. In the pressure history, it can be observed that the pressure is reduced from 9 to 4.4 bar during the pressure equalization–depressurization step and 4.4 to 0.3 bar during the blowdown step, leading to the desorption of methane and carbon dioxide. In the purge step, some H_2_ ends up in the heavy product, besides desorbed CO_2_ and CH_4_, because part of the light product (*i.e.*, syngas stream) is fed to the column. Afterward, the pressure increases to 2 and 9 bar in the pressure equalization–pressurization and counter-pressurization steps, respectively, which improves CO_2_ and methane adsorption capacity.

**Figure 13:**
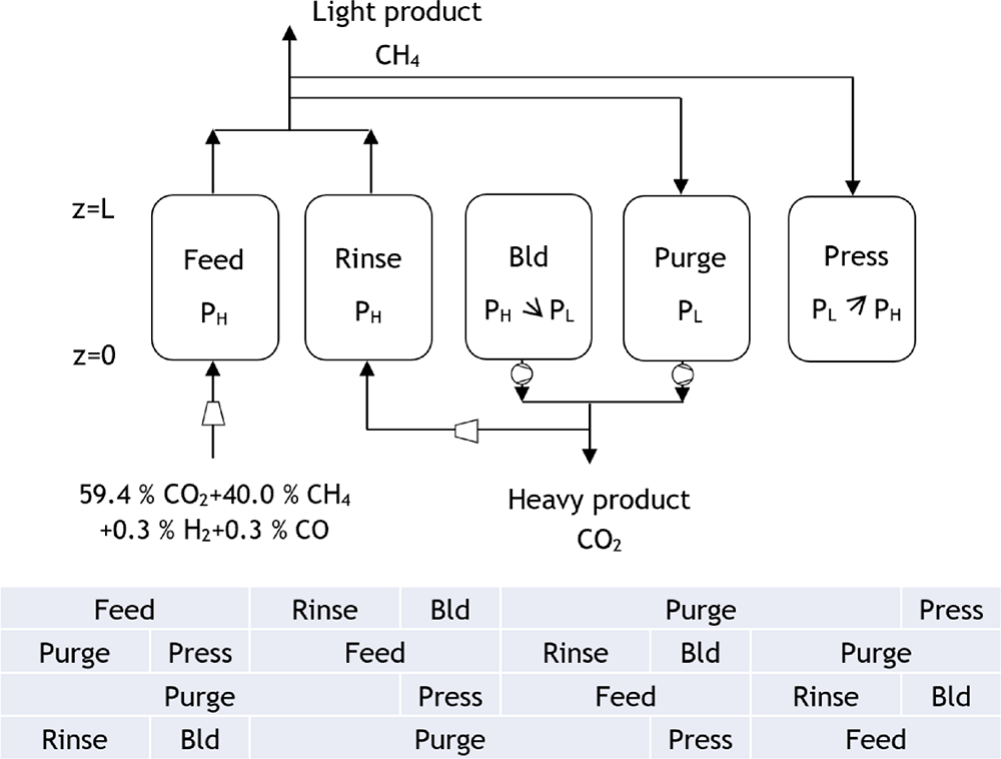
Proposed cycle for the PSA 2 of case study 2.

To obtain methane and carbon dioxide with high purity, the heavy product generated in PSA 1, which contains 59.4% of CO_2_, 40.0% of CH_4_, 0.3% of H_2_, and 0.3% of CO, was compressed and fed at 343 K and 9 bar and with a molar flow rate of 2089 mol h^–1^ to a second PSA placed in series with the first one. The proposed cycle for PSA 2 of case study 2 and the corresponding extension for a four-column unit are presented in Figure 13. This PSA cycle comprises the following five steps: feed, rinse, counter-current blowdown, purge, and counter-current pressurization. For the extension to a four-column unit, the time of the blowdown step must be equal to the time for the pressurization step, and the sum of the duration of the rinse and blowdown steps has to be the same as the one for the feed step. A feed step time of 435 s and a rinse step of 230 s were defined. Therefore, the total cycle time is 1740 s.

The adsorption bed characteristics and the operating conditions used in the mathematical model of PSA 2 for this case study are presented in Table 8. The transport parameters used in the simulation of this PSA are summarized in Table S5.

**Table 8:** Adsorption Bed Characteristics and Operating Conditions Used in the Simulations of PSA 2 for Case Study 2

bed characteristics	
bed length (m)	3
internal bed diameter (m)	0.4
bed porosity	0.37
operating conditions	
*T* (K)	343
*P*_H_ (bar)	9
*P*_L_ (bar)	0.35
*y*_in, feed_	CO_2_: 0.594; CH_4_: 0.400; H_2_: 0.003; CO: 0.003
*F*_in_ (mol h^–1^)	feed: 2089; rinse: 1985; purge: 12.2; press: 2442
step times (s)	feed: 435; rinse: 230; Bld: 205; purge: 665; press: 205

**Figure 14:**
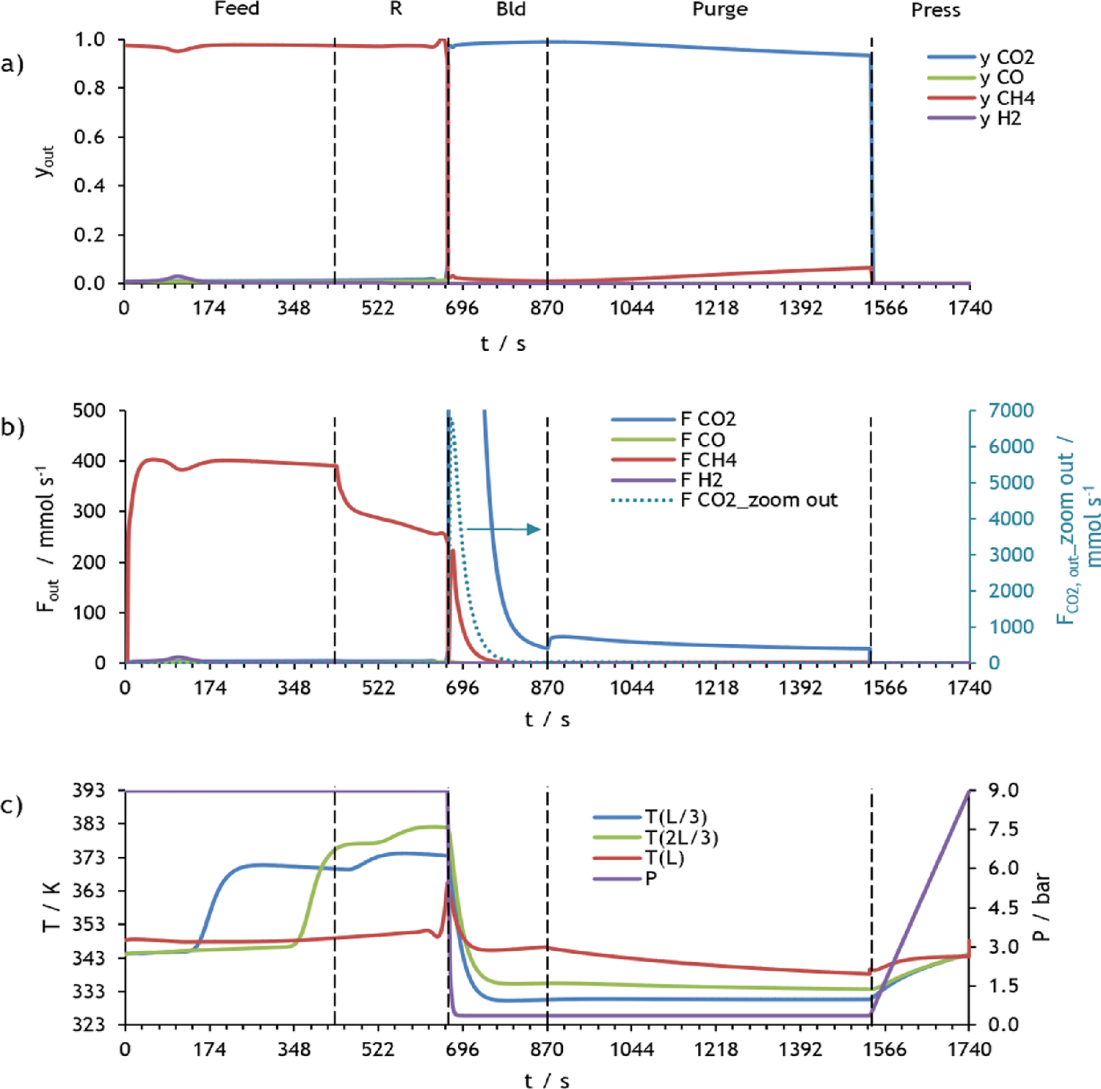
Simulation results at CSS for PSA 2 of case study 2: (a) molar fraction of each component at the column outlet, (b) molar flow rate of each component at the column outlet, and (c) pressure and temperature at three different positions within the column.

The molar fraction and molar flow rate of the components of the mixture, as well as pressure and temperature values attained during one cycle in CSS for the second PSA of case study 2, are shown in Figure 14. This figure shows that all carbon dioxide is adsorbed during feed plus rinse time, resulting in the production of a methane stream with high purity. Afterward, the blowdown step decreases the bed pressure from 9 to 0.35 bar, which leads to the adsorbent regeneration; *i.e.*, CO_2_ desorbs and elutes to the column outlet. The column continues to be regenerated during the purge step, in which a fraction of the light product is fed counter-currently at low pressure. So, a high-purity CO_2_ product is generated during blowdown plus purge times. In the pressurization step, the pressure increases from 0.35 to 9 bar, and thus, CO_2_ starts to adsorb once again.

The performance of a cyclic adsorption process is typically measured according to product purity, product recovery, and adsorbent productivity.^25^ For case study 1, the performance parameters are described by:
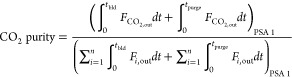
4
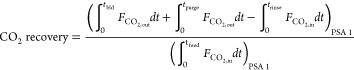
5
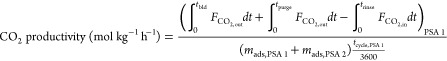
6
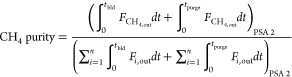
7
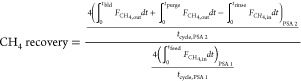
8
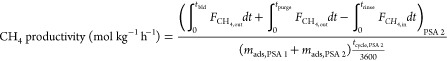
9
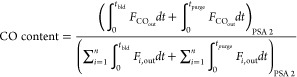
10
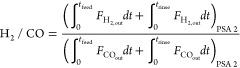
11
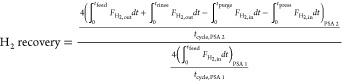
12
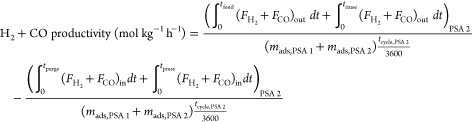
13The performance parameters for case study 2 are defined by the following:
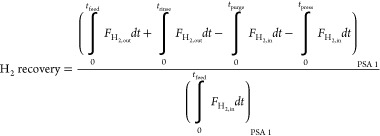
14
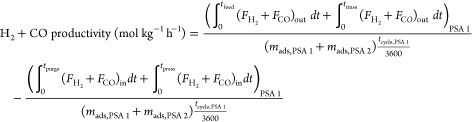
15
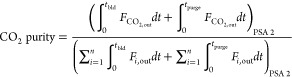
16
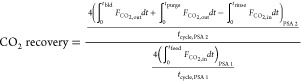
17
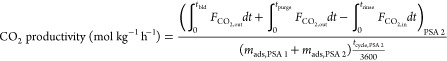
18
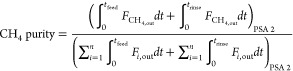
19
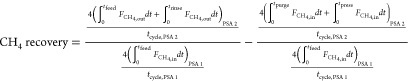
20
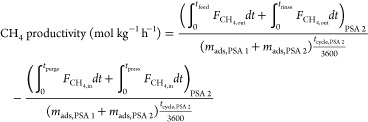
21
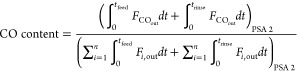
22

The power consumption of the process is obtained by eq 23, considering adiabatic compression^25^ and that the CO_2_ electroreduction reactor operates at the inlet feed temperature of the dual-PSA process (*i.e.*, 343 K):
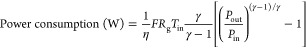
23where η is the efficiency; *F* is the total molar flow rate; *T*_in_ is the inlet temperature; *P*_in_ and *P*_out_ are the inlet and outlet pressure, respectively; and γ is the ratio between the gas mixture molar specific heat at constant pressure and the one at constant volume. For compression of the feed and rinse streams, an efficiency of 85% is assumed. In the case of the vacuum pumps required for the blowdown and purge steps, an efficiency of 60% is considered.

On the other hand, the power consumption of the first PSA for each dual-PSA process taking into account adiabatic compression^25^ and that the CO_2_ electroreduction reactor and the dual-PSA unit operate at different temperatures is determined by eq 24:
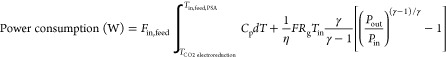
24where *F*_in, feed_ is the inlet total molar flow rate of the feed step, *T*_CO2 electroreduction_ is the operating temperature of the CO_2_ electroreduction reactor, *T*_in, feed, PSA_ is the inlet feed temperature of each dual-PSA process (= 343 K), and *C*_p_ is the gas mixture molar specific heat at constant pressure. The value of the energy consumption of each dual PSA considering heating of the feed inlet stream of the first PSA was obtained for a temperature of the CO_2_ electroreduction reactor equal to the room temperature (∼298 K).

The dual-PSA performance obtained in the cyclic simulations for case studies 1 and 2 is presented in Table 9. According to this table, the proposed PSA process for case study 1 allows the production of CO_2_ with a purity higher than 97% and a recovery of around 95%. However, it does not satisfy the required methane specifications; *i.e.*, it produces methane with low purity (90.5%) despite the high recovery that can be obtained (95.2%). Although 97% CH_4_ purity is not achieved, the value of 90.5% is high enough to be injected into the natural gas grid in some European countries such as France. Additionally, the CO content in the methane stream, which is vital to quantify because this compound can harm human health, is lower than 2%. Moreover, the H_2_/CO ratio of the syngas product is about 4.7, which means that a CO adjustment must be performed before it can be used in the Fischer–Tropsch process. The productivities of CH_4_, CO_2_, and H_2_ + CO for the dual PSA for case study 1 are 0.39, 0.58, and 0.48 mol kg_ads_^–1^ h^–1^, respectively. In contrast, the proposed industrial PSA for case study 2 enables the production of both methane and carbon dioxide products with high purities and recoveries (>97 and 95%, respectively) while achieving a CO content in the methane stream inferior to 1%. Therefore, unlike the first configuration, the second one meets the required specifications for the CH_4_ and CO_2_ products despite demanding an energy consumption about two times higher (64.9 vs 29.8 W h mol_CH4_^–1^). Taking into account the heating of the feed inlet stream of each dual-PSA process from room temperature to 343 K, the energy consumption is around 36.6 W h mol_CH4_^–1^ for case study 1 and 71.6 W h mol_CH4_^–1^ for case study 2. In addition, the H_2_/CO ratio of the syngas product is around 4.4, and so a CO adjustment must be carried out before it can be utilized in the Fischer–Tropsch process. The productivities of CH_4_, CO_2_, and H_2_ + CO for the dual PSA for case study 2 are 0.39, 0.60, and 0.45 mol kg_ads_^–1^ h^–1^, respectively; thus, both case studies obtained similar productivities for the three products.

**Table 9:** Dual-PSA Performance for Case Studies 1 and 2

	case study 1	case study 2
purity (%)	CH_4_: 90.5; CO_2_: 99.1	CH_4_: 97.5; CO_2_: 97.3
CO content in the CH_4_ stream (%)	1.8	0.8
H_2_/CO	4.7	4.4
recovery (%)	CH_4_: 95.2; CO_2_: 95.1; H_2_: 96.7	CH_4_: 95.3; CO_2_: 98.7; H_2_: 97.9
productivity (mol kg_ads_^–1^ h^–1^)	CH_4_: 0.39; CO_2_: 0.58; H_2_ + CO: 0.48	CH_4_: 0.39; CO_2_: 0.60; H_2_ + CO: 0.45
energy consumption (W h mol_CH4_^–1^)	29.8	64.9
energy consumption considering heating the feed inlet stream of the dual PSA (W h mol_CH4_^–1^)	36.6	71.6

## 4 Conclusions

A multistep PSA process to obtain high-purity carbon dioxide, methane, and syngas products resulting from the CO_2_ electroreduction reaction has been proposed. To this end, molecular simulations were combined with PSA simulations to analyze the separation performance of zeolites for a representative mixture composed of 40%_v_ of CO_2_, 27%_v_ of CH_4_, 27%_v_ of H_2_, and 6%_v_ of CO. A set of adsorbents with varying pore sizes, topologies, and chemical compositions was initially investigated using Monte Carlo and molecular dynamics simulations. On the basis of the adsorption equilibrium of the pure components and their mixtures, NaX and MFI zeolites have been selected because of their affinity for CO_2_ and CH_4_, respectively. These two zeolites have been utilized to design and simulate a dual-step PSA process that maximizes the purity and recovery of the main products. So, CH_4_ should present a purity above 97% to be used as domestic gas, CO_2_ should be recovered with high purity to be recycled back into the electroreduction unit for maximization of its CO_2_ conversion, and syngas should have a H_2_/CO ratio preferably around 2 to be used in the Fischer–Tropsch process.

In this work, two alternative configurations for the dual PSA were studied, with the order in which the separation of CO_2_/CH_4_/syngas occurs being their main difference. In case study 1, the CO_2_ contained in the feed mixture is retained in the NaX zeolite in a first PSA, and then methane is separated from the syngas stream in a second PSA using the MFI zeolite. The simulation results showed that the proposed dual-PSA process for case study 1 is capable of obtaining CO_2_ with high purity and recovery (99.1 and 95.1%, respectively) but produces methane with a purity below the required one for it to be used as domestic gas (90.5 vs 97%) in spite of the high methane recovery that is achieved (95.2%). It can also be concluded that the produced methane can be instead injected into the natural gas grid in some European countries (*e.g.*, France and Netherlands). Moreover, this process produces a syngas product with a H_2_/CO ratio of around 4.7, and thus, an adjustment with CO in the syngas product must be carried out before it can be fed to a Fischer–Tropsch process. The obtained productivities of CH_4_, CO_2_, and H_2_ + CO for this case study are 0.39, 0.58, and 0.48 mol kg_ads_^–1^ h^–1^, respectively. As for case study 2, a mixture mostly composed of CO_2_ and methane is separated from the syngas stream in a first PSA with the use of the MFI zeolite, and afterward, CO_2_ is removed from methane using NaX zeolite in a second PSA. It is possible to conclude that the methane and CO_2_ products can be obtained in the proposed dual-PSA process for case study 2 with purity above 97% and a high recovery (*i.e.*, >95% for CH_4_ and >98% for CO_2_). Besides, a H_2_/CO ratio of syngas of around 4.4 is achieved, which means that a CO adjustment must be accomplished prior to the syngas product being utilized in the Fischer–Tropsch process. Therefore, this case study meets the required methane specifications for it to be used as domestic gas. However, a much higher value for the energy consumption of this process (64.9 W h mol_CH4_^–1^) is observed when compared to the proposed process of case study 1 (29.8 W h mol_CH4_^–1^). If the heating of the feed inlet stream of the dual-PSA process from room temperature to 343 K is considered, then the energy consumption is around 36.6 W h mol_CH4_^–1^ for the first case study and 71.6 W h mol_CH4_^–1^ for the second. Additionally, a CH_4_ productivity of 0.39 mol kg_ads_^–1^ h^–1^, a CO_2_ productivity of 0.60 mol kg_ads_^–1^ h^–1^, and a H_2_ + CO productivity of 0.45 mol kg_ads_^–1^ h^–1^ can be achieved in this last case study.

## Supporting Information Available

Standard reduction potentials of the reduction half-reactions vs the normal hydrogen electrode (NHE) at a pH of 7, scheme of the CO_2_ electroreduction reaction, representative structure of the set of zeolites under study and their pore size distribution, adsorption equilibrium isotherms for pure components as well as for the ternary (CH_4_: 0.45; H_2_: 0.45; CO: 0.1) and quaternary (CO_2_: 0.40; CH_4_: 0.27; H_2_: 0.27; CO: 0.06) mixtures, adsorption equilibrium selectivity of the components for the ternary and quaternary mixtures, mean squared displacements for pure components at infinite dilution on the zeolites, large cavity diameter and pore limiting diameter of the zeolites, and transport parameter values used in the simulations (PDF)
