# Supplementary material for: An Efficient Strategy for Electroreduction Reactor Outlet Fractioning into Valuable Products
Source: Ind Eng Chem Res. 2023 May 26;62(22):8847–63. doi: 10.1021/acs.iecr.3c00090 (PMC10251741; doi:10.1021/acs.iecr.3c00090)
Supplement: Supplementary file 1 — ie3c00090_si_001.pdf [file ie3c00090_si_001.pdf]

An efficient strategy for electro-reduction reactor outlet fractioning into valuable products

SUPPORTING INFORMATION

Mariana C. N. Bessa[1,2,*], *Azahara Luna-Triguero*[3,4], *Jose M. Vicent-Luna*[5,*], *Paulo M. O. C. Carmo*[1,2], *Mihalis N. Tsampas*[6], *Ana Mafalda Ribeiro*[1,2], *Alírio E. Rodrigues*[1,2], *Sofia Calero*[4,5], *Alexandre F. P. Ferreira*[1,2]

[1] Laboratory of Separation and Reaction Engineering - Laboratory of Catalysis and Materials (LSRE-LCM), Department of Chemical Engineering, University of Porto, Rua Dr. Roberto Frias, s/n, 4200-465 Porto, Portugal

[2] ALiCE - Associate Laboratory in Chemical Engineering, Faculty of Engineering, University of Porto, Rua Dr. Roberto Frias, 4200-465 Porto, Portugal

[3] Energy Technology, Department of Mechanical Engineering, Eindhoven University of Technology, P.O. Box 513, 5600 MB Eindhoven, The Netherlands

[4] Eindhoven Institute for Renewable Energy Systems (EIRES), Eindhoven University of Technology, P.O. Box 513, Eindhoven 5600 MB, The Netherlands

[5] Materials Simulation and Modelling, Department of Applied Physics and Science Education, Eindhoven University of Technology, 5600 MB Eindhoven, The Netherlands

[6] Dutch Institute For Fundamental Energy Research (DIFFER), 5612AJ Eindhoven, the Netherlands

*Corresponding author: up201503607@edu.fe.up.pt; j.vicent.luna@tue.nl

Table S1. Thermodynamic potentials of the reduction half-reactions vs. NHE, at a pH = 7 [1]

Reduction half-reactions	Thermodynamic potential, E_0 (V) vs. NHE
$CO_2 + e^- \rightleftharpoons CO_2^{\bullet -}$	-1.90
$CO_2 + 2H^+ + 2e^- \rightleftharpoons HCOOH$	-0.61
$CO_2 + 2H^+ + 2e^- \rightleftharpoons CO + H_2O$	-0.53
$CO_2 + 4H^+ + 4e^- \rightleftharpoons HCHO + H_2O$	-0.48
$CO_2 + 6H^+ + 6e^- \rightleftharpoons CH_3OH + H_2O$	-0.38
$CO_2 + 8H^+ + 8e^- \rightleftharpoons CH_4 + 2H_2O$	-0.24
$2H^+ + 2e^- \rightleftharpoons H_2$	-0.41

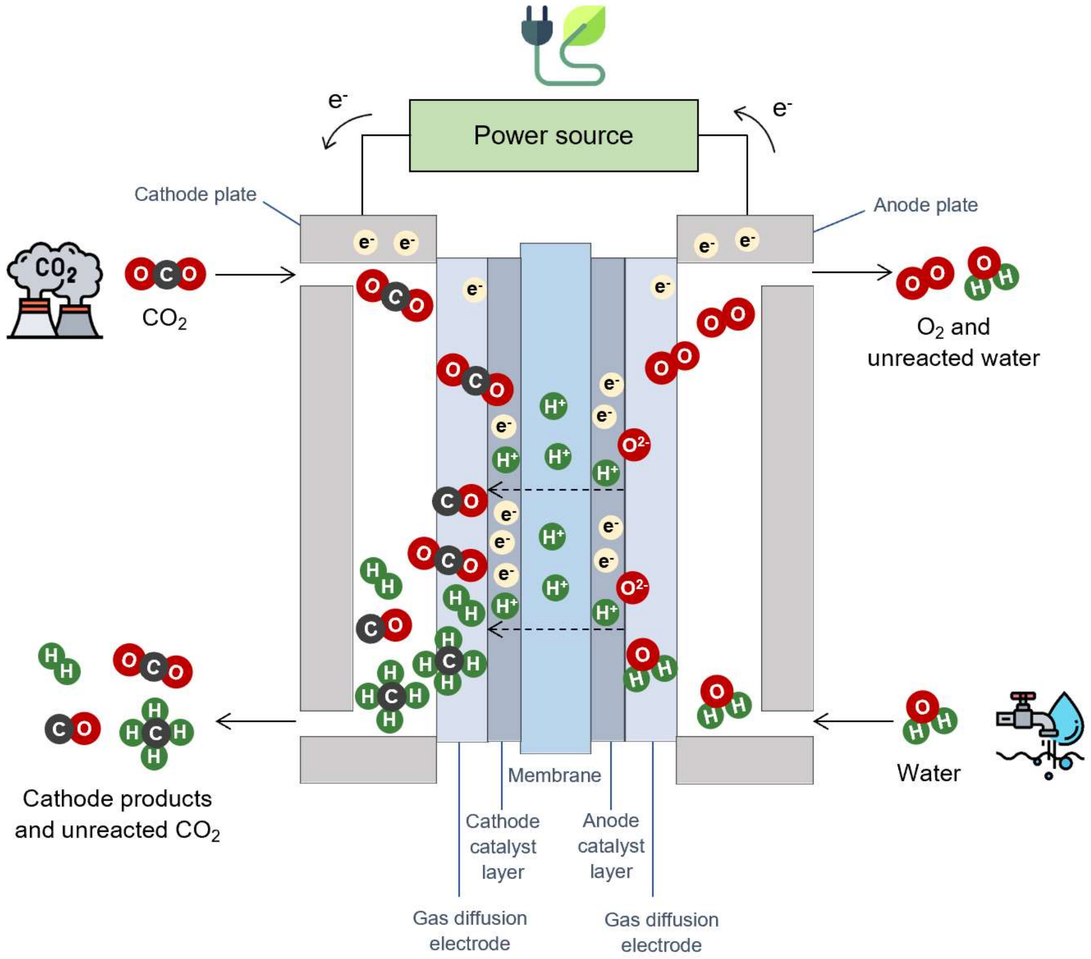

Figure S1. Scheme of the CO_2 electroreduction reaction.

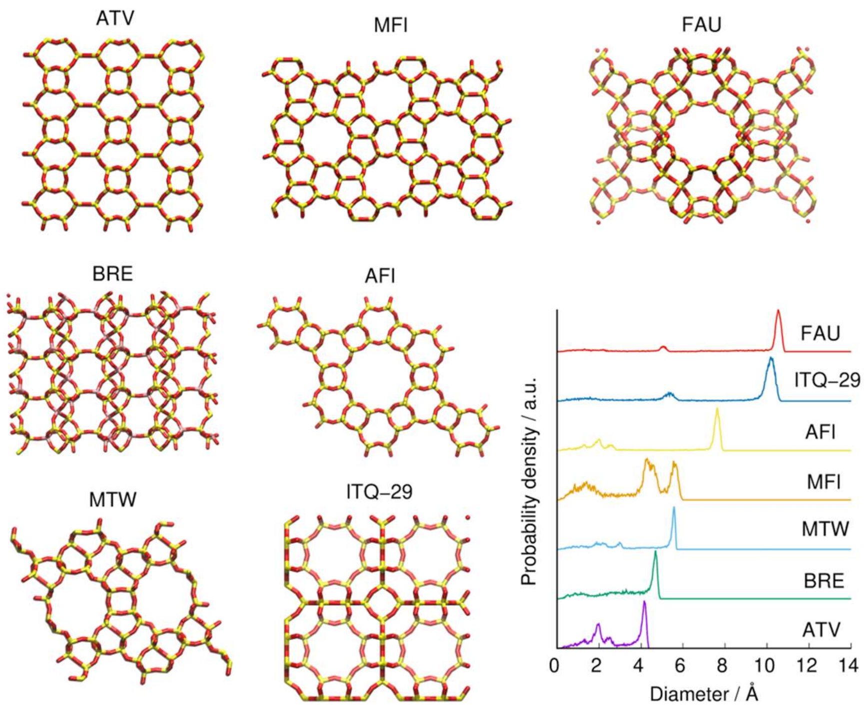

Figure S2. Representative structure of the selected zeolites and their pore size distribution (PSD). FAU topology comprises HS-FAU, NaY, and NaX zeolites.

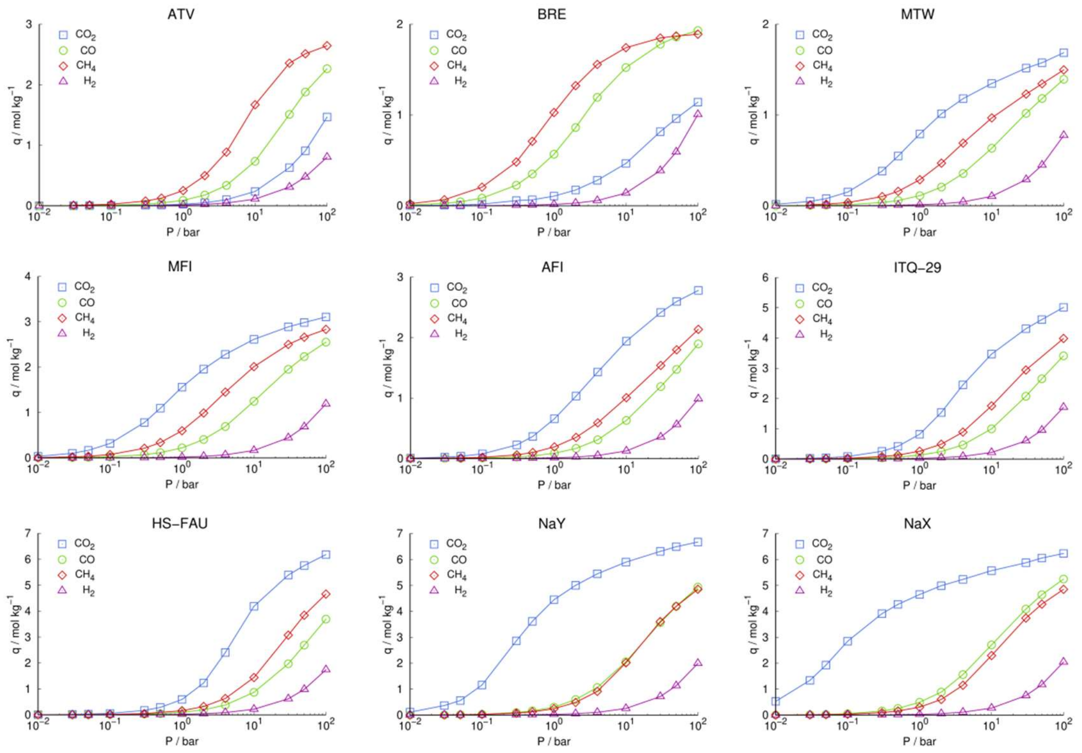

Figure S3. Adsorption equilibrium isotherms for pure components CO_2, CO, CH_4, and H_2 on the selected zeolites at 308 K and up to 100 bar.

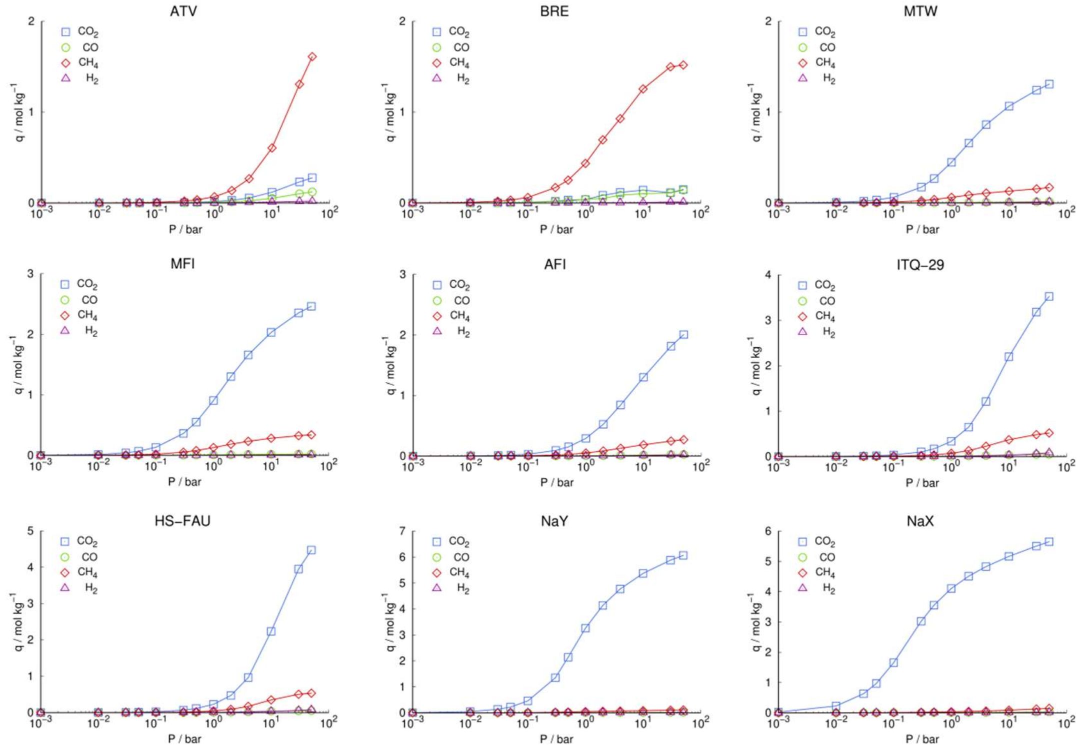

Figure S4. Adsorption equilibrium isotherms for quaternary mixture (CO_2: 0.40; CH_4: 0.27; H_2: 0.27; CO: 0.06) on the selected zeolites at 308 K and up to 100 bar.

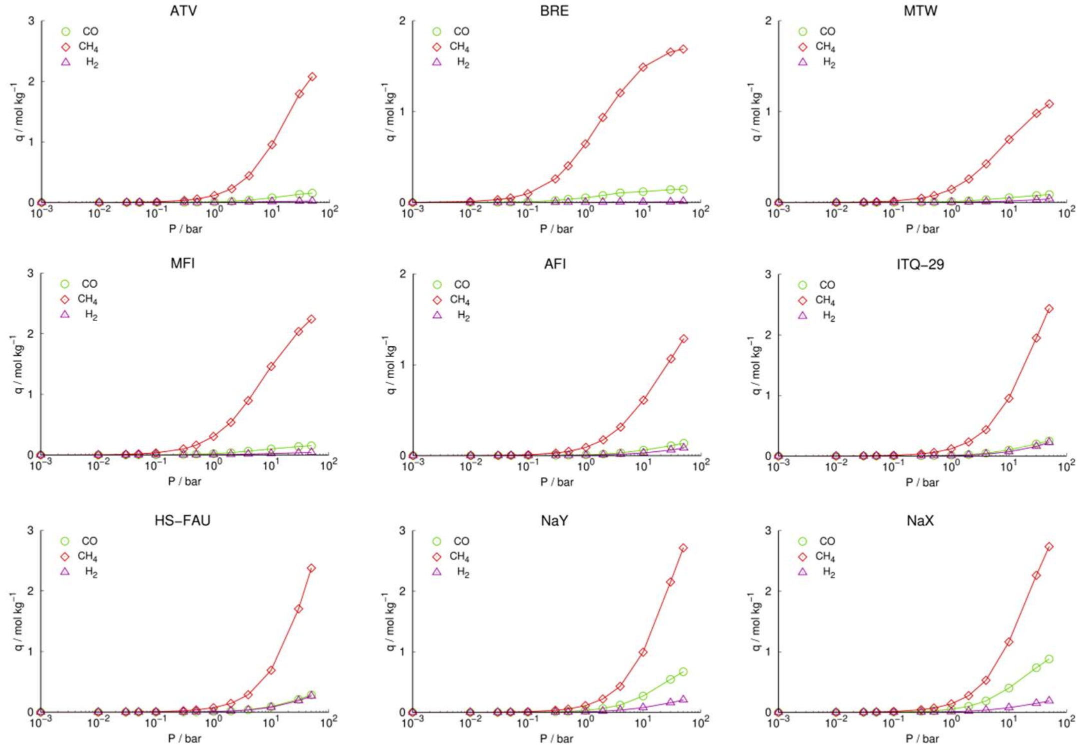

Figure S5. Adsorption equilibrium isotherms for ternary mixture (CH_4: 0.45; H_2: 0.45; CO: 0.1) on the selected zeolites at 308 K and up to 100 bar.

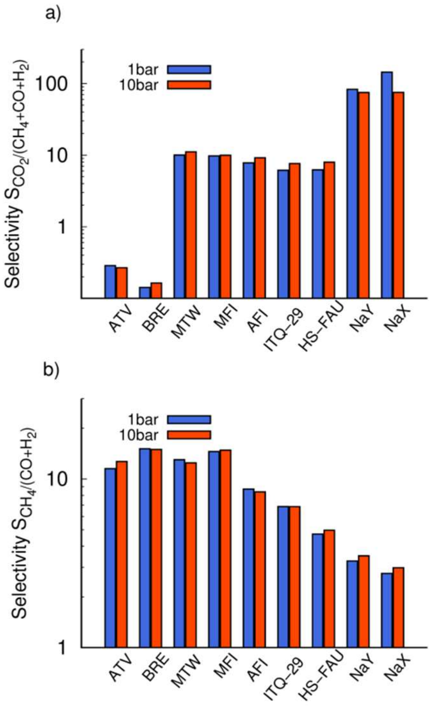

Figure S6. Adsorption equilibrium selectivity for: a) CO_2 over CH_4, CO, and H_2 corresponding to the quaternary mixture (CO_2: 0.40; CH_4: 0.27; H_2: 0.27; CO: 0.06); b) CH_4 over CO and H_2 corresponding to the ternary mixture (CH_4: 0.45; H_2: 0.45; CO: 0.1) on the selected zeolites at 308 K and 1 and 10 bar.

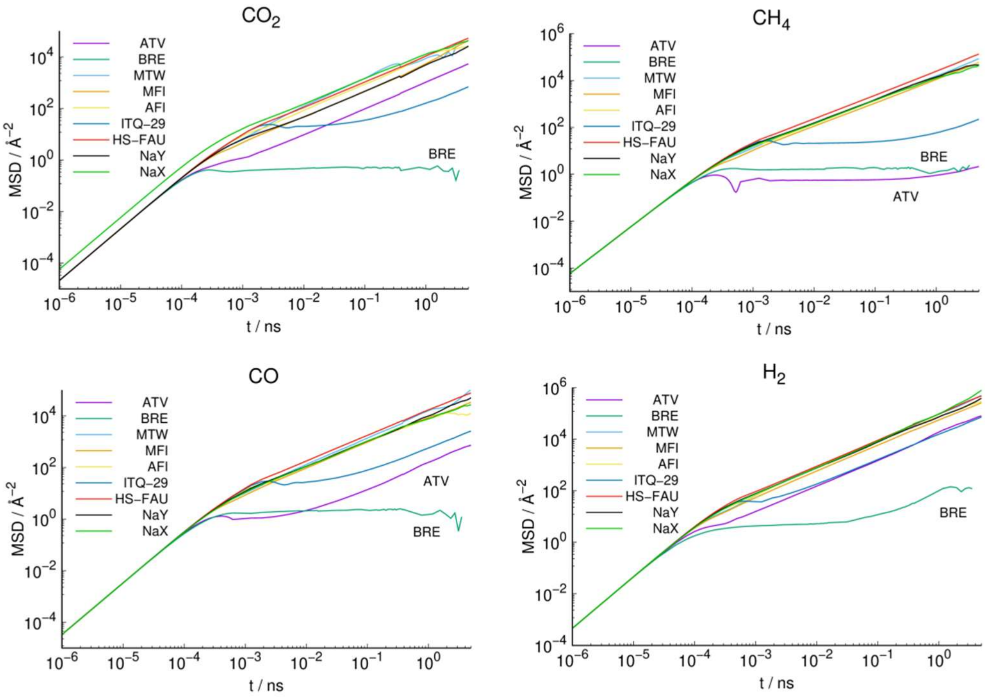

Figure S7. Mean squared displacements (MSD) for pure components CO_2, CO, CH_4, and H_2 at infinite dilution on the selected zeolites at 373 K.

Table S2. Large cavity diameter and pore limiting diameter (PLD) of the zeolites. FAU topology comprises HS-FAU, NaY, and NaX zeolites. PLD is estimated form the peak of the PSD (**Figure S2**) while LCD is taken from the IZA database

Zeolite	LCD (Å)	PLD (Å)
**ATV**	4.2	3.48
**BRE**	4.7	2.96
**MTW**	5.7	5.68
**MFI**	5.8	4.7
**AFI**	7.6	7.42
**ITQ-29**	10.2	4.21
**FAU**	10.5	7.35

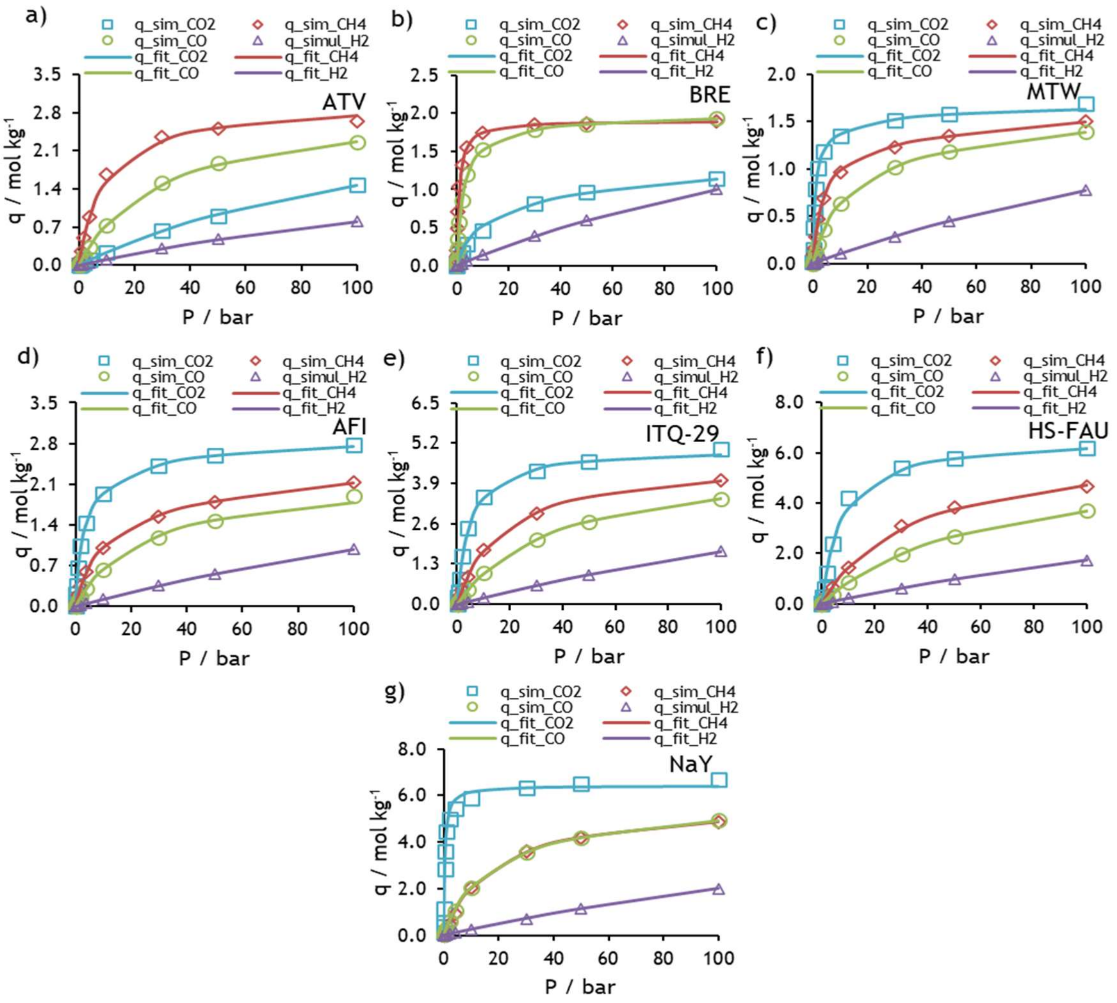

Figure S8. Adsorption equilibrium data and corresponding DSL fit for CO_2, CO, CH_4, and H_2 as pure components on a) ATV, b) BRE, c) MTW, d) AFI, e) ITQ-29, f) HS-FAU, and g) NaY, at 308 K and up to 100 bar.

Table S3. DSL parameters for CH_4, CO_2, CO, and H_2 using ATV, BRE, MTW, AFI, ITQ-29, HS-FAU, and NaY zeolites

	b_1 (bar^{-1})	$q_{sat,1}$ (mol kg^{-1})	b_2 (bar^{-1})	$q_{sat,2}$ (mol kg^{-1})
**ATV**				
H_2	1.4 x 10^{-4}	2.3	4.6 x 10^{-3}	2.5
CH_4	6.7 x 10^{-2}	0.43	0.11	2.6
CO	3.3 x 10^{-2}	2.9	1.2 x 10^{-5}	0.72
CO_2	7.6 x 10^{-3}	3.3	6.4 x 10^{-3}	0.10
**BRE**				
H_2	4.9 x 10^{-3}	2.8	3.9 x 10^{-4}	2.4
CH_4	1.4	1.0	0.92	0.87
CO	0.49	1.7	4.3 x 10^{-2}	0.30
CO_2	1.6 x 10^{-2}	0.91	0.19	0.61
**MTW**				
H_2	5.2 x 10^{-4}	1.7	4.2 x 10^{-3}	2.3
CH_4	5.5 x 10^{-4}	4.3	0.27	1.3
CO	3.8 x 10^{-3}	0.9	9.0 x 10^{-2}	1.3
CO_2	4.8 x 10^{-2}	0.4	1.3	1.3
**AFI**				
H_2	3.4 x 10^{-3}	3.8	1.4 x 10^{-4}	3.0
CH_4	0.11	1.8	3.3 x 10^{-3}	1.8
CO	4.1 x 10^{-2}	2.2	5.7 x 10^{-3}	0.11
CO_2	0.45	1.9	5.2 x 10^{-2}	1.0
**ITQ-29**				
H_2	1.6 x 10^{-3}	4.4	3.9 x 10^{-3}	4.0
CH_4	8.6 x 10^{-2}	1.0	5.1 x 10^{-2}	3.7
CO	2.7 x 10^{-2}	4.7	1.3 x 10^{-3}	0.12
CO_2	0.28	0.49	0.20	4.6
**HS-FAU**				
H_2	3.0 x 10^{-3}	7.0	4.5 x 10^{-4}	3.2
CH_4	8.8 x 10^{-3}	0.57	3.1 x 10^{-2}	5.9
CO	2.2 x 10^{-2}	1.5	1.5 x 10^{-2}	4.4
CO_2	0.13	4.4	0.15	2.3
**NaY**				
H_2	8.6 x 10^{-4}	5.4	3.8 x 10^{-3}	5.6
CH_4	2.0 x 10^{-4}	1.7	5.4 x 10^{-2}	5.7
CO	6.7 x 10^{-2}	4.7	1.1 x 10^{-2}	1.6
CO_2	2.4	3.5	2.1	2.9

Table S4. Transport parameters used in the simulations for case study 1

	**PSA 1**	**PSA 2**
D_{ax} (m^2 s^{-1})	3.15×10^{-4}	4.47×10^{-4}
λ (W m^{-1} K^{-1})	0.497	0.658
k_f (m s^{-1})	1.51×10^{-2}	2.38×10^{-2}
h_f (W K^{-1} m^{-2})	149	245
h_w (W K^{-1} m^{-2})	59.6	47.2
U (W m^{-2} K^{-1})	27.2	24.3

[a] Values at feed inlet conditions.

Table S5. Transport parameters used in the simulations for case study 2

	**PSA 1**	**PSA 2**
D_{ax} (m^2 s^{-1})	3.03×10^{-4}	1.73×10^{-4}
λ (W m^{-1} K^{-1})	0.450	0.351
k_f (m s^{-1})	1.88×10^{-2}	9.35×10^{-3}
h_f (W K^{-1} m^{-2})	185	98.1
h_w (W K^{-1} m^{-2})	59.6	61.8
U (W m^{-2} K^{-1})	27.2	27.7

[a] Values at feed inlet conditions.

REFERENCES

(1) Duarah, P.; Haldar, D.; Yadav, V. S. K.; Purkait, M. K. Progress in the electrochemical reduction of CO2 to formic acid: A review on current trends and future prospects. *J Environ Chem Eng* **2021**, *9* (6).
